# Single‐Cell Analysis Reveals that Vitamin C Inhibits Bone Metastasis of Renal Cancer via Cell Cycle Arrest and Microenvironment Remodeling

**DOI:** 10.1002/advs.202501011

**Published:** 2025-05-28

**Authors:** Jianye Zhang, Qi Zhang, Gang Lin, Ying Wang, Juan Li, Ping Wang, Jie Qi, Yuan Liang, Shiming He, Yanqing Gong, Ninghan Feng, Yang Wang, Yuanyuan Ma, Mei Zhang, Yue Shi, Xuesong Li, Weimin Ci, Liqun Zhou

**Affiliations:** ^1^ Department of Urology Peking University First Hospital Beijing 100034 P. R. China; ^2^ Institute of Urology Peking University Beijing 100034 P. R. China; ^3^ National Urological Cancer Center Beijing 100034 P. R. China; ^4^ China National Center for Bioinformation Beijing 100101 P. R. China; ^5^ Beijing Institute of Genomics Chinese Academy of Sciences Beijing 100101 P. R. China; ^6^ Department of Thoracic Surgery Peking University First Hospital Peking University Beijing 100034 P. R. China; ^7^ Department of Urology Affiliated Wuxi No. 2 Hospital of Nanjing Medical University Wuxi 214002 P. R. China; ^8^ Animal Center Peking University First Hospital Beijing 100034 P. R. China; ^9^ Department of Urology Chinese PLA General Hospital Beijing 100039 P. R. China; ^10^ Department of Urology The First Affiliated Hospital of Henan University Kaifeng 475001 P. R. China

**Keywords:** APM, bone metastasis, microenvironment, renal cancer, treatment

## Abstract

Bone metastasis is the second most common site of distant metastatic spread in renal cell carcinoma (RCC) patients, significantly contributing to cancer‐related mortality. The metastatic process is driven by both intrinsic tumor cell properties, such as cancer stem cell‐like characteristics, and the bone microenvironment. Understanding the complex interactions between cancer cells and their niche is crucial for identifying therapeutic targets to eliminate metastasis‐initiating cells and prevent overt metastasis. In this study, a murine bone metastasis model is developed using renal cancer cells derived from fibrin gel‐induced 3D tumor spheres, which exhibit stem‐like phenotypes. It is found that a stable form of vitamin C, L‐ascorbic acid 2‐phosphate sesquimagnesium (APM), significantly inhibits the growth of renal cancer stem‐like cells in vitro and the progression of RCC bone metastasis in vivo. Single‐cell RNA sequencing revealed that APM induces cell cycle arrest and reduces the metastatic potential of cancer cells. Furthermore, APM remodels the tumor microenvironment by suppressing osteoclast differentiation and neutrophil recruitment. Combining APM with a CXCR2 antagonist, SB225002, further inhibits bone metastasis progression. This study provides a high‐resolution profile of vitamin C's antitumor effects in the bone metastatic microenvironment and supports the rationale for clinical trials of vitamin C in bone metastatic RCC.

## Graphical abstract

3D‐cultured renal cancer cells exhibit stem‐like phenotypes and are more efficient in generating bone metastases. Vitamin C treatment can suppress the development of bone metastasis. Single‐cell transcriptomic analysis reveals that vitamin C inhibits metastasis by inducing tumor cell cycle arrest, suppressing osteoclast differentiation, and reducing neutrophil recruitment.

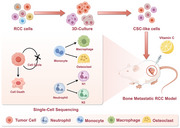

## 1 Introduction

Bone is the second most common site of distant metastatic spread (following the lung) in 30% of patients with advanced renal cell carcinoma (RCC).^[^ ^1^ ^]^ This metastatic process significantly contributes to cancer‐related mortality, often leading to severe skeletal‐related events such as severe pain, hypercalcemia, nerve compression, and pathologic fractures.^[^ ^2^ ^]^ Substantial progress has been made in the medical management, including surgery, radiotherapy, targeted therapy, and combination therapy, of bone metastasis.^[^ ^1a^ ^]^ However, these treatments are only palliative, and the outcomes remain unsatisfactory. Therefore, developing effective strategies to manage and treat RCC metastasis is an urgent clinical need.

Bone metastasis is a multistage process and depends on both tumor‐intrinsic traits and various molecules supplied by the bone environment, a concept that can be intuitively explained by the “seed and soil” hypothesis. Bone is not the only and final destination of metastatic cells. A series of analyses have demonstrated frequent “metastasis‐to‐metastasis” seeding.^[^ ^3^ ^]^ At least two‐thirds of bone metastases can subsequently seed secondary metastases in other organs, leading to patient death.^[^ ^4^ ^]^ Bone micrometastases actually caused when diagnosed as nonbone organ metastases, possibly because cancer cells initially arrive in the bone and acquire more aggressive characteristics and are thus further induced to establish more overt metastases in both bones and other organs.^[^ ^5^ ^]^ Studies have shown that disseminated tumor cells in bone marrow exhibit cancer stem cell‐like properties, which may contribute to the reactivation of dormant cells, entry into the cell cycle, and the formation of multiorgan micrometastases.^[^ ^5^, ^6^ ^]^ Therefore, therapies that target cancer stem cell‐like cells directly could potentially prevent metastasis.

While the molecular mechanisms of cancer metastasis are well understood in some cancers,^[^ ^7^ ^]^ effective treatments to eliminate cancer cells in the bone niche remain lacking. Developing strategies to prevent bone micrometastases from progressing to more overt metastases in both bone and nonbone organs is crucial.^[^ ^8^ ^]^ Animal models are important tools for investigating the pathogenesis and developing treatment strategies for bone metastases. However, the limited availability of RCC animal models necessitates the development of new in vivo approaches to design effective therapies.

Vitamin C is an essential nutrient for humans and with known antitumor properties. Clinical studies have shown that vitamin C is a safe and effective treatment for many advanced cancer patients.^[^ ^9^ ^]^ Our previous research demonstrated that a stable form of vitamin C, L‐ascorbic acid 2‐phosphate sesquimagnesium (APM), inhibits the growth of clear cell renal cell carcinoma (ccRCC) cells both in vitro and in vivo.^[^ ^10^ ^]^ Additionally, APM has been shown to inhibit the proliferation of cancer stem‐like cells in prostate cancer cell lines.^[^ ^11^ ^]^ Another in vivo study showed that vitamin C promotes the osteoblast differentiation effect and reduces the osteoclast differentiation potential of marrow cells.^[^ ^12^ ^]^ Combined treatment with vitamins C and D₃ significantly inhibits RCC tumor growth.^[^ ^13^ ^]^ However, the role of APM in controlling cancer cells during bone metastasis and its potential for combination with conventional drugs remain largely unexplored.

In this study, we established a robust bone metastasis model using Renca cells obtained from soft fibrin gel‐induced 3D tumor spheres,^[^ ^14^ ^]^ which exhibit cancer stem cell‐like phenotypes and are more efficient in generating metastases than 2D‐cultured cells. Through Ingenuity Pathway Analysis (IPA) of bulk RNA‐seq data, we found that vitamin C‐related pathways were significantly downregulated in the 3D cultured tumor cells. Treatment with APM effectively killed renal cancer stem‐like cells in vitro and significantly inhibited bone metastasis in vivo. To evaluate molecular and tumor immune microenvironment‐related features accompanying APM treatment, single‐cell transcriptomic analysis revealed dynamic changes in cell percentages, subtype heterogeneity, and intercellular interactions among malignant, immune, and stromal cells following APM treatment. Furthermore, APM with a CXCR2 antagonist, SB225002, further inhibited bone metastasis progression. These findings provide deep insights into the mechanisms of APM in metastasis prevention and therapy, supporting the therapeutic potential of vitamin C in cancer treatment.

## 2 Results

### 2.1 3D‐Cultured Renal Cancer Cells Exhibit Cancer Stem‐Like Features and Increased Metastatic Potential

**Figure 1:**
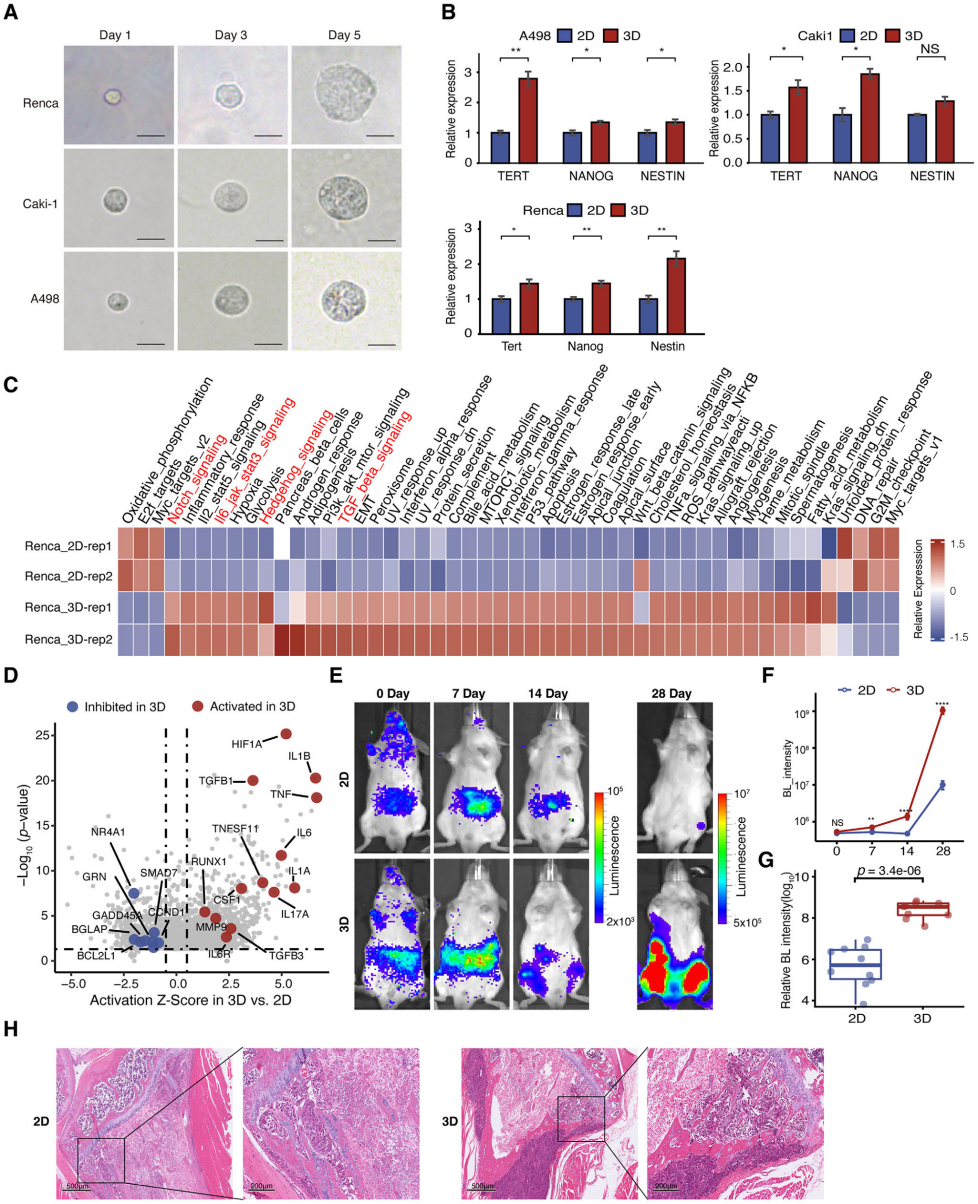
The 3D‐cultured renal cancer cells were similar to cancer stem cells and efficiently formed bone metastases. A) A single A498 cell, Caki‐1 cell and Renca cell grew into a multicellular tumor spheroid within a 90 Pa 3D fibrin gel during the culture time course from Day 1 to Day 5. Bar, 50 µm. B) The mRNA levels of stem cell markers in 2D and 3D cultures of Renca, A498, and Caki‐1 were quantified by real‐time quantitative polymerase chain reaction (RT‒qPCR). The error bars indicate the means ± SDs. The *p* values were determined by Student's *t* test; *p<0.05, **p<0.01, ***p<0.001, ****p<0.0001. C) Heatmap of transcriptome analysis results indicating stem cell‐associated and malignancy‐associated signaling pathways upregulated in 3D‐cultured cells. D) Volcano plot showing the results of Ingenuity Pathway Analysis (IPA) to predict differences in upstream regulators between the 3D‐cultured and 2D‐cultured group of Renca cells. E) 2D‐ and 3D‐cultured Renca/luc cells were used to establish a mouse model of bone metastasis (2D group, *n*=10; 3D group, *n*=9), showing increased metastatic burden in 3D‐cultured groups at 28 days post‐injection. F) Growth curve obtained via BL in the region of interest for comparing the 3D group and 2D group, demonstrating enhanced tumor growth in 3D group. The p values were determined by Student's *t* test; *p<0.05, **p<0.01, ***p<0.001, ****p<0.0001. G) Box plot showing the differences in the *ex vivo* BL imaging intensity between 3D group and the 2D group, with significant differences observed in the 3D group. H) HE staining of tumor sections, revealing increased tumor burden and extracortical invasion in 3D‐cultured groups.

Stem cell‐like cancer cells are a highly tumorigenic subpopulation that play crucial roles in tumor initiation, promotion, and metastasis.^[^ ^15^ ^]^ We utilized a well‐established 3D fibrin gel culture system^[^ ^14^ ^]^ to generate aggressive cancer stem‐like cells by culturing renal cancer cells in fibrin matrices with a stiffness of ≈90 Pa. Mouse Renca RCC cells and human A498 and Caki‐1 RCC cells were trapped individually in the gel and grew into a spheroid‐like morphology after 5 days of culture (**Figure** **1**A). These 3D‐cultured RCC cells exhibited upregulated expression of stem cell markers, including *NANOG*, *NESTIN*, *TERT*, *CD24*, *ALDH1A3*, and *ALDH1A1*, compared to 2D‐rigid‐dish‐cultured control cells (Figure 1B; Figure S1A, Supporting Information). To investigate the transcriptional changes in these stem‐like cancer cells, we performed bulk RNA sequencing (RNA‐seq) analysis on both 3D‐ and 2D‐cultured cells. The 3D‐cultured cells showed upregulation of many stemness‐ and metastasis‐related signaling pathways, such as Notch, Hedgehog, epithelial‐mesenchymal transition (EMT), and TGF‐β pathways. Notably, inflammatory signaling pathways, including IL‐6 and JAK‐STAT3 pathways, were also activated in the 3D‐cultured groups (Figure 1C; Figure S1B,C, Supporting Information). Using IPA, we identified potential upstream regulators in 3D‐cultured Renca, Caki‐1 and A498 cells, such as *TGF‐β1*, *HIF‐1a*, *IL‐1b*, *IL‐6*, and *MMP9* (Figure 1D; Figure S1D,E and Tables S1 and S2, Supporting Information), which are strongly associated with metastasis.^[^ ^16^ ^]^ Integration our transcriptome data of 3D‐cultured Renca cells with a published study,^[^ ^17^ ^]^ and confirmed significant upregulation of *TGF‐β1* in 3D‐cultured Renca cells (Figure S1F, Supporting Information). Similar result was observed in 3D‐cultured A498 and Caki‐1 cells (Figure S1G, Supporting Information). Treatment with the TGF‐β Type I Receptor inhibitor SB431542 significantly inhibited tumor sphere growth in a dose‐dependent manner (Figures S1H–M, Supporting Information). While these findings highlight the critical role of *TGF‐β1* in cancer stem cell growth, therapeutic targeting of *TGF‐β1* may pose challenges due to potential toxicity concerns at high concentrations of SB431542. Therefore, alternative pathways that could offer safer and more clinically applicable therapeutic are needs.

We further established a mouse model of bone metastasis using 3D‐ and 2D‐cultured Renca/luc cells injected into the caudal artery (CA).^[^ ^18^ ^]^ After 28 days, 100% of mice injected with 3D‐cultured Renca/luc cells (3D group) developed bone metastasis, compared to 60% in the 2D group. In vivo bioluminescence (BL) imaging showed significantly higher BL intensities in the hind limbs of 3D group mice (Figure 1E,F; Figure S1N, Supporting Information). Moreover, the growth curve derived from BL imaging indicated stronger tumor signals in 3D group mice, with significant growth observed after 14 days (Figure 1F). After 28 days, bone tissues of the two groups were separate, ex vivo BL intensity also confirmed higher metastatic burden in the 3D group (Figure 1G). Hematoxylin‐eosin (HE) staining revealed that the tumor burden in the femur of 3D group mice was higher and invaded outside of the bone cortex, contrasting with the more limited burden in the 2D group (Figure 1H). Collectively, these findings demonstrate that renal cancer cells cultured in 3D fibrin gels exhibit cancer stem‐like phenotypes and enhanced metastatic potential.

### 2.2 Vitamin C Inhibits the Growth of 3D‐Cultured Renal Cancer Cells and the Formation of Bone Metastasis

**Figure 2:**
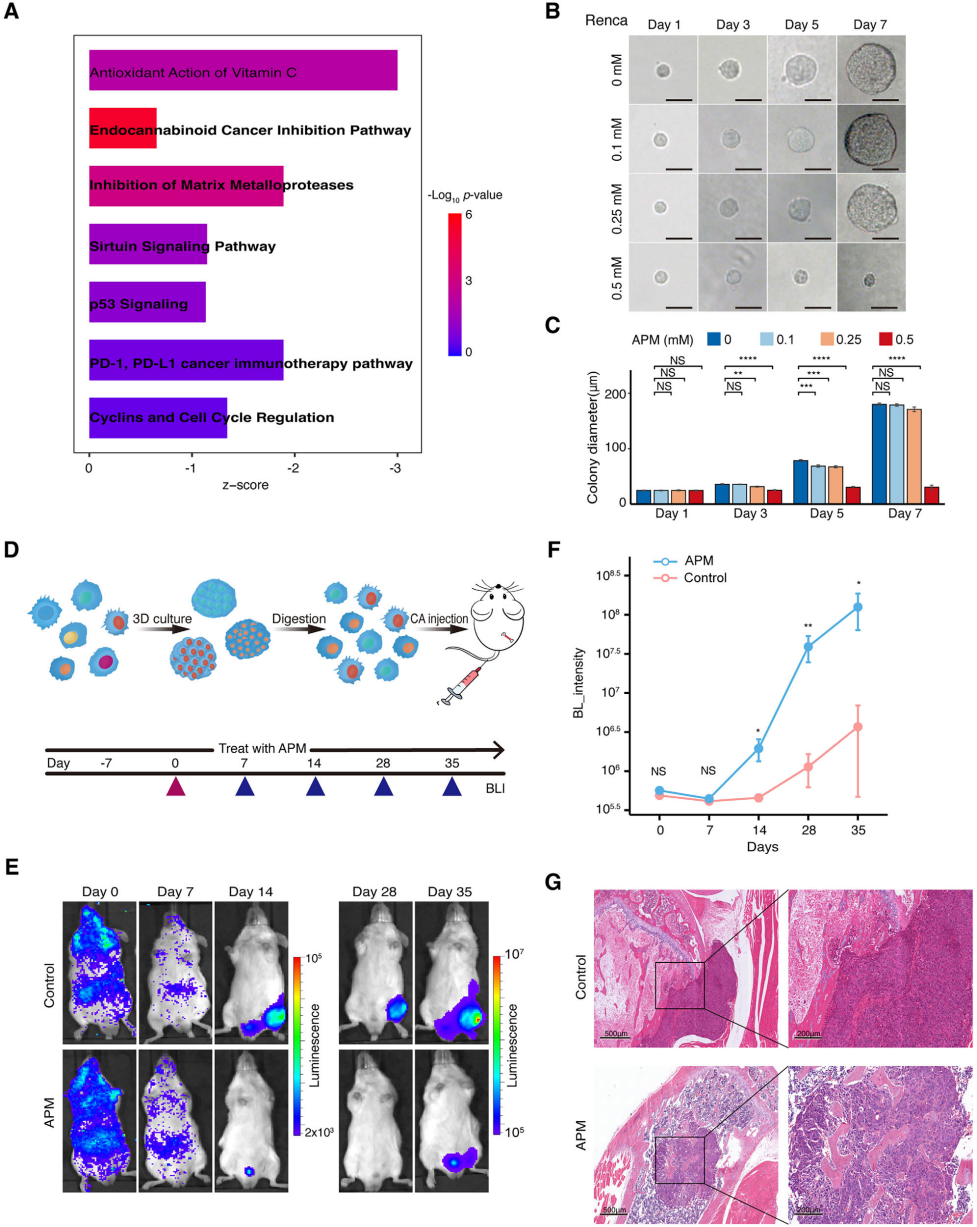
L‐ascorbic acid 2‐phosphate sesquimagnesium (APM) inhibited the growth of 3D‐cultured Renca cells in vitro and the progression of bone metastasis in vivo. A) IPA analysis predicting the downregulation of pathways related to the vitamin C pathway, cell cycle regulation, and PD1/PD‐L1 cancer immunotherapy in 3D‐cultured cells. B) APM treatment significantly inhibits the growth of 3D tumor spheres derived from Renca cells in vitro. Bar, 50 µm. C) Dose‐dependent inhibition of 3D tumor sphere growth upon APM treatment. The error bars indicate the means ± SDs. The *p* values were determined by Student's *t* test; *p < 0.05, **p < 0.01, ***p < 0.001, and ****p < 0.0001. D) Schematic of the APM treatment protocol in mice with bone metastatic RCC. E) Representative BL images showing reduced tumor burden in APM‐treated mice compared to controls at multiple time points. (APM group, *n* =7; control group, *n*=8). F) Growth curves from BL in the region of interest for comparing the APM group and control group, demonstrating the inhibitory effect of APM on tumor growth. The error bars indicate the means ± SDs. The *p* values were determined by Student's *t* test; *p < 0.05, **p < 0.01, ***p < 0.001, and ****p < 0.0001. G) HE staining was performed to compare the difference tumor burden in the bone marrow between the two groups.

To elucidate the mechanism underlying the maintenance of the cancer stem cell‐like phenotype, we conducted upstream regulator analysis using IPA. Notably, the antioxidant activity of the vitamin C pathway was significantly suppressed in 3D‐cultured renal cancer cells, as were cell cycle regulation and PD1/PD‐L1 cancer immunotherapy pathways (**Figure** **2**A; Figure S2A,B, and Tables S1 and S2, Supporting Information). Our previous study demonstrated that the vitamin C derivative APM, exhibits reduced nonspecific cell damage and enhanced stability in mouse plasma compared to vitamin C.^[^ ^10^ ^]^ This led us to hypothesize that vitamin C could effectively inhibiting the growth of 3D‐cultured renal cancer cells and potentially prevent bone metastases of RCC. Consistent with our hypothesis, APM treatment significantly impaired the growth of 3D tumor spheres derived from Renca, A498 and Caki‐1 cells (Figure 2B,C; Figure S2C–F, Supporting Information). To assess the efficacy of APM in inhibiting bone metastasis in vivo, we first pretreated wild‐type BALB/c mice with APM (0.5 g kg^−1^) or normal saline (control) via intraperitoneal injection for 7 days (once daily), followed by the injection of 3D‐cultured Renca/luc cells. Mice were then treated daily with APM (0.5 g kg^−1^) or normal saline until the endpoint at 5 weeks (Figure 2D). APM treatment markedly reduced the metastatic rate and growth of RCC bone metastases compared to control mice (APM: 3/7 mice vs control: 6/8 mice) (Figure 2E,F; Figure S2G, Supporting Information). Histological examination via hematoxylin‐eosin (HE) staining revealed a decreased renal cancer cell burden in the bone marrow of APM‐treated mice (Figure 2G). Collectively, these results suggest that APM effectively inhibits the growth and metastatic potential of renal cancer cells.

### 2.3 Single‐Cell Transcriptome Analysis Reveals that Vitamin C Induces Cell Cycle Arrest and Downregulates Prometastatic Signaling

To further investigate the impact of APM on the tumor cell community in RCC bone metastases, we generated single‐cell RNA sequencing (scRNA‐seq) profiles of 33 912 high‐quality transcriptomes in APM‐treated (*n* = 13 212) and nontreated (*n* = 20 700) bone metastatic samples after quality control and filtering (Figure S3A, Supporting Information). We identified14 major cell types based on classical marker genes expression, including tumor cells, endothelial cells, fibroblasts, chondrocytes, osteoclasts, osteoblasts, monocytic cells, neutrophils, T cells, B cells, natural killer (NK)/NKT cells, plasmacytoid dendritic cells (pDCs), pericytes and erythroid cells (Figure S3B–D and Table S3, Supporting Information). Notably, the relative abundance of osteoclasts and neutrophils was higher in APM‐naïve cells compared to APM‐treated cells, while B and NK/NKT cells were less abundant (Figure S3E, Supporting Information). Moreover, among the cell populations, tumor cells, monocytic cells (including monocytes, macrophages, osteoclasts and DCs) and neutrophils expressed higher levels of vitamin C receptors (Figure S3F, Supporting Information), suggesting these cells are particularly responsive to vitamin C.

**Figure 3:**
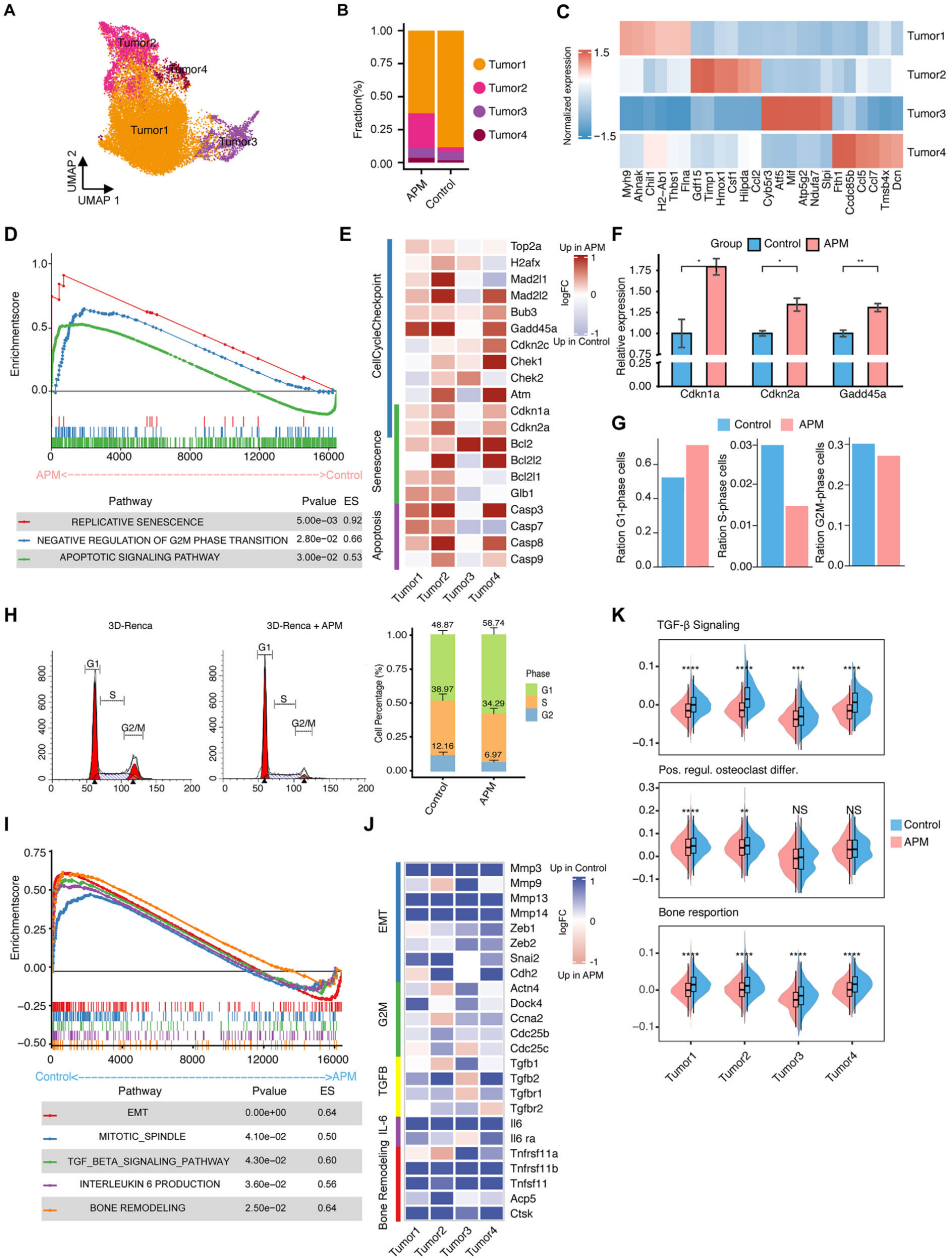
Identification and characterization of malignant cells in bone metastasis model mice treated with APM. A) Uniform Manifold Approximation Projection (UMAP) plots of subclustered malignant cells, colored and labeled by cluster. B) Bar plots showing the average proportions of tumor subtypes in response to APM treatment and control group. C) Heatmap showing the expression of selected marker genes in malignant cell subtypes. D) Gene Set Enrichment Analysis (GSEA) revealing the upregulation of pathways associated with replicative senescence, negative regulation of G2/M phase transition and apoptotic in APM‐treated tumor cells. E) Heatmap showing the upregulation of cell cycle checkpoint‐, cell senescence‐ and apoptosis‐related genes in APM‐treated tumor cells. F) RT‐qPCR validation of cell cycle checkpoint markers in 3D‐cultured Renca cells with and without APM treatment. The error bars indicate the means ± SDs. The *p* values were determined by Student's *t* test; *p < 0.05, **p < 0.01, ***p < 0.001, and ****p < 0.0001. G) Bar plot showing the proportions of G1‐phase, S‐phase and G2/M‐phase cycling malignant cells in APM‐treated and APM‐naïve samples. H) Flow cytometry analysis confirming the accumulation of cells in G1 phase and reduction in S and G2/M phases upon APM treatment. The percentages of cells in each phase of the cell cycle were quantified. The experiments were repeated in triplicate, and the results are presented as the means. I) GSEA of the EMT, mitotic spindle, interleukin‐6 production and bone remodeling signatures showing downregulation in APM‐treated malignant cells. J) Heatmap of gene expression changes associated with EMT‐, mitotic spindle‐, IL‐6 signaling‐ and bone remodeling‐related. K) Violin and box plots comparing the positive regulation of osteoclast differentiation and bone resorption signature score distributions between APM‐treated and untreated groups. The *p* values were determined by a two‐sided Wilcoxon rank‐sum test; *p < 0.05, **p < 0.01, ***p < 0.001, and ****p < 0.0001.

We further evaluate the effect of APM on tumor cells in RCC bone metastases by reclustering malignant cells based on epithelial markers (*Krt8* and *Krt18*) (Figure S4A, Supporting Information), identifying four subtypes (Tumors 1, 2, 3, and 4) with different proportions between the APM‐treated and APM‐naïve groups (**Figure** **3**A–C; Figure S4B, Supporting Information). Gene set enrichment analysis (GSEA) revealed activation of replicative senescence, negative regulation of G2/M phase transition, and cell apoptotic signaling pathways in APM‐treated tumor cells compared to APM‐naïve cells (Figure 3D; Figure S4C, Supporting Information). The expression of cell cycle checkpoint‐, cell senescence‐ and apoptosis‐related genes, such as *Cdkn1a* (p21), *Cdkn2a* (p16) and *Gadd45a*, were upregulated in APM‐treated cancer cells of the four subtypes (Figure 3E), and this finding was further validated by real‐time quantitative polymerase chain reaction (RT‒qPCR) in Renca (Figure 3F), A498 and Caki‐1 cells (Figure S4D, Supporting Information). Using the cyclone function and MKI67 expression, we categorized cell into non‐cycling and cycling status including G1, S and G2M phase (Figure S4E,F, Supporting Information). APM‐treated tumor cells scored higher for mitotic cell cycle arrest and lower for cell cycle phase transition gene sets (Figure S4G, Supporting Information). Moreover, the APM‐treated group showed accumulation of cancer cells in G1 phase and a subsequent decrease in the proportions of S‐ and G2/M‐phase cells (Figure 3G), which was further confirmed by flow cytometric analysis in 3D‐cultured Renca (Figure 3H), A498 (Figure S4H, Supporting Information) and Caki‐1 (Figure S4I, Supporting Information) cells treated with APM. The same results were observed in the tumor cells of bone metastatic mice which were treat with APM the proportion of G2M were significant decreased (Figure S4J, Supporting Information).

Furthermore, we identified several bone metastasis‐related pathways, including EMT, IL‐6 production, and bone remodeling, and their associated genes were significantly downregulated in APM‐treated malignant cells compared with APM‐naïve cells (Figure 3I–K; Figure S5A, Supporting Information). A lower proportion of *Mmp13^+^*, *Tnfsf11^+^*, and *Il‐6^+^* malignant cells was observed in APM‐treated samples (Figure S5B, Supporting Information). These genes, *Mmp13*,^[^ ^19^ ^]^ *Tnfsf11* (RANKL)^[^ ^20^ ^]^ and *Il‐6*,^[^ ^21^ ^]^ are central to the interactions between tumor and bone cells, promoting osteoclast‐induced bone degradation and osteoclast differentiation. RT‐qPCR validation showed significantly decreased expression levels of *Mmp13*, *Il‐6*, and *Tnfsf11* in APM‐treated A498, Caki‐1, and Renca cells (Figure S5C, Supporting Information). Intriguingly, APM‐treated tumor cells in mice exhibited downregulated expression of the chemokine *CXCL2*, known to contribute to bone osteolysis in metastatic cancer,^[^ ^22^ ^]^ and downregulation of the CXCL2 production pathway (Figure S5A,D, Supporting Information). Collectively, these single‐cell resolution results indicate that APM suppresses tumor growth by inducing cell cycle arrest in malignant cells and interfering with prometastatic signaling pathways.

### 2.4 Vitamin C Mediates Transcriptomic Shift in Stromal Cells and Monocytic Cells in Bone Metastatic RCC

Given the negative enrichment of bone remodeling and IL‐6 production signaling pathways in APM‐treated malignant cells, we speculated that APM plays a significant role in reprogramming stromal and myeloid subpopulation in bone metastases. Osteoblasts, a component of the stromal cell population derived from mesenchymal stem cells,^[^ ^23^ ^]^ we analyzed via unsupervised clustering of stromal cells and obtained five distinct clusters—fibroblasts, osteoblasts, chondrocytes, pericytes and endothelial cells in both APM‐treated and APM‐naïve groups (Figure S6A,B, Supporting Information). GSEA revealed upregulation of negative regulation of osteoclast differentiation and downregulation of EMT, TGF‐β, IL‐1‐mediated signaling, IL‐6 production and neutrophil chemotaxis signaling pathways in stromal cells of the APM‐treated group compared to the APM‐naïve group (Figure S6C, Supporting Information). Moreover, APM‐treated stromal cells exhibited suppressed osteoclast differentiation, characterized by low expression of cytokines such as *Mmp3*, *Mmp13*, *Il‐1a*, *Il‐1b*, *Il‐6*, and *Tnfsf11*, and downregulated chemokines including Cxcl1, Cxcl2, Cxcl3, and Cxcl5, leading to reduced neutrophil recruitment (Figure S6D, Supporting Information).

**Figure 4:**
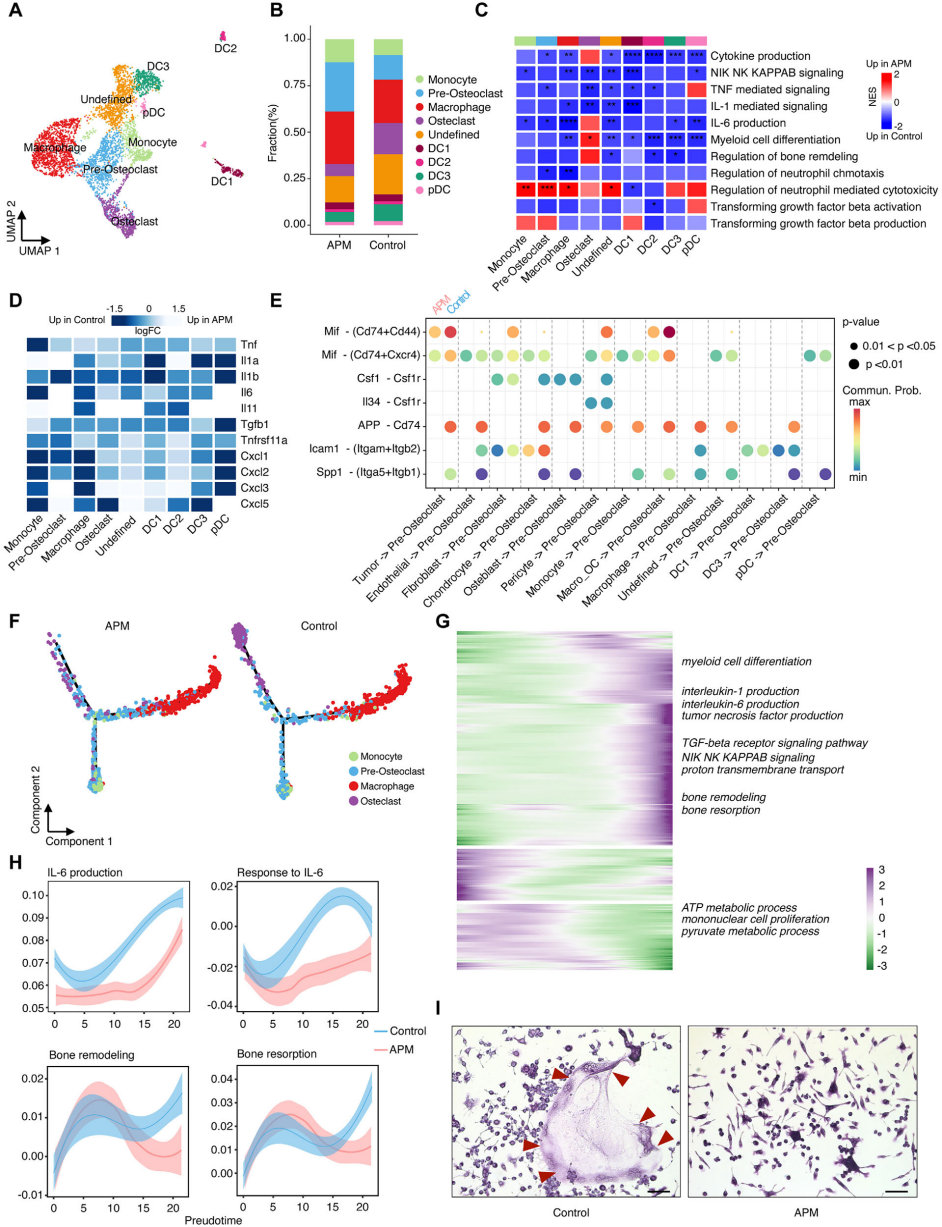
Vitamin C mediates transcriptomic shifts in stromal and monocytic cells in bone metastatic RCC. A) UMAP plot of myeloid cells captured across all samples, colored and labeled by cell type. B) Bar plots showing the average proportions of myeloid cell subtypes in response to APM treatment and control group. C) Heatmap of GSEA scores indicating the pathways significantly enriched in myeloid subtypes from APM‐treated samples and APM‐naïve samples. D) Heatmap indicating showing the downregulation of cytokine genes in APM‐treated myeloid cells. E) The significant ligand‒receptor pairs that contribute to the signals sent from other cell groups to preosteoclasts. The dot color and size indicate the calculated communication probability and p value, respectively. F) Pseudotime trajectory analysis of monocytes, preosteoclasts, macrophages, and osteoclasts from APM‐treated and APM‐naïve samples. Cell subtypes are labeled by colors. G) Heatmap showing the dynamic changes in gene expression along the pseudotime axis. H) 2D plots showing the expression scores for genes related to IL‐6 production, the response to IL‐6, bone remodeling and bone resorption in APM‐treated (red) and APM‐naïve (blue) samples along with the pseudotime trajectory. I) Trap staining demonstrating the inhibitory effect of APM on osteoclast differentiation of murine macrophage cell line RAW264.7. The formation of multinucleated TRAP‐positive osteoclasts is significantly reduced in the presence of APM‐treated cells. Arrow showed the cells of osteoclast differentiation. Bar, 50 µm.

In monocytic cells population, nine clusters were identified: monocytes (*Vcan* ^+^), osteoclast precursor cells (Preosteoclasts; *Pf4* ^+^), macrophages (*Apoe* ^+^), osteoclasts (*Acp5* ^+^), DC1s (*Ccl22* ^+^), DC2s (*Clec9a* ^+^), DC3s (*Cd209a* ^+^), and pDCs (*Siglech* ^+^) (**Figure** **4**A,B; Figure S6E,F, Supporting Information). GSEA indicated upregulation of neutrophil‐mediated cytotoxicity and downregulation of TNF‐mediated signaling, IL‐1‐mediated signaling, IL‐6 production, myeloid cell differentiation, regulation of bone remodeling and regulation of neutrophil chemotaxis in APM‐treated cells compared to APM‐naïve cells (Figure 4C), and their associated genes were downregulated (Figure 4D). A focused analysis of the myeloid compartment revealed substantial population shifts between the APM‐treated and APM‐naïve fractions: with APM‐treated cells predominantly consisting of immature preosteoclasts and a decreased relative percentage of mature osteoclasts (Figure 4B). This was corroborated by the abundance of interactions related to osteoclast differentiation (Mif‐Cd74+Cd44, Mif‐Cd74+Cxcr4, App‐Cd74, and Csf1‐Csfr1) in the APM‐naïve group (Figure 4E). The continuous developmental trajectory of monocytes, preosteoclasts, macrophages and osteoclasts, as determined by Monocle2, showed a binary branched structure with monocytes differentiated into macrophages and osteoclasts via stepwise pathways passing through osteoclast precursor cells (Figure S6G, Supporting Information). Preosteoclasts were predominantly found in APM‐treated samples, with fewer mature osteoclasts identified at the end of the cell state transition path, contrasting with the APM‐naïve samples that had more mature osteoclasts at the terminal ends (Figure 4F). Pathway analysis associated with these transition states indicated upregulation of IL‐6 production, bone remodeling and resorption at the late stage, with these gene signatures being more pronounced in APM‐naïve cells compared to APM‐treated cells (Figure 4G,H; Figure S6H,I, Supporting Information). These finding were validated in vitro using the murine macrophage cell line RAW264.7, where TRAP stain showed significantly decreased osteoclast differentiation upon APM treatment (Figure 4I).

Furthermore, considering thar tumor inflammatory monocytes (TIMs) and tumor‐associated macrophages (TAMs) are implicated in bone metastasis and associated with a worse prognosis.^[^ ^24^ ^]^ We assessed our data for TIM and TAM subsets by scoring single cells for their respective gene signatures. APM‐naïve cells exhibited significantly stronger TIM and TAM signatures (Figure S6J, Supporting Information), TIM and TAM associated marker genes were also highly expression (Figure S6K, Supporting Information). These results suggest that APM treatment may inhibit osteoclast differentiation and impair RCC bone metastasis.

### 2.5 Vitamin C Inhibits the CXCL‐CXCR2 Signaling Axis in Neutrophils to Reduce Bone Metastasis

**Figure 5:**
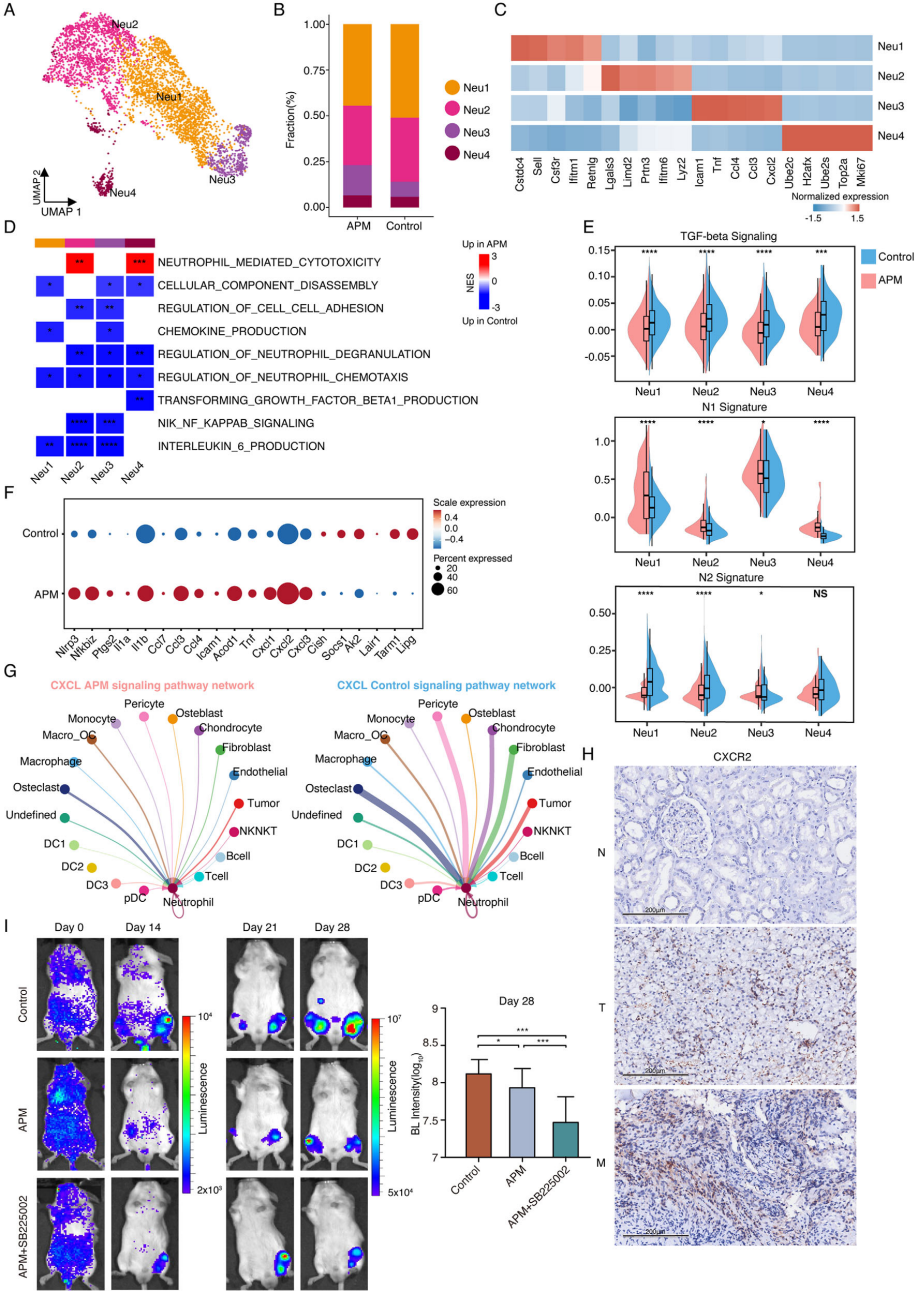
Vitamin C inhibits the CXCL‐CXCR2 signaling axis in neutrophils to reduce bone metastasis. A) UMAP plot of neutrophils subtypes, revealing distinct clusters in APM‐treated and untreated samples, colored and labeled by cell type. B) Bar plots showing the average proportions of neutrophil subtypes in response to APM treatment and control group. C) Heatmap showing the expression of selected marker genes in neutrophil subtypes. D) Heatmap showing the upregulation of pathways associated with TGF‐β signaling, NIF NK kappa signaling, and IL‐6 production in APM‐naïve neutrophils, and upregulation of neutrophil mediated cytotoxicity in APM‐treated neutrophils. E) Violin and box plots comparing the TGF‐β signaling, N1‐like neutrophil and N2‐like neutrophil signature score distributions among neutrophil subtypes by treatment status. The *p* values were determined by a two‐sided Wilcoxon rank‐sum test; *p < 0.05, **p < 0.01, ***p < 0.001, ****p < 0.0001. F) Dot plot comparing the expression of representative marker genes for N1 and N2 neutrophil subtypes between APM and control groups. APM treatment is associated with higher expression of N1 markers, while control groups exhibit higher expression of N2 markers. The size of the dots corresponds to the percentage of cells expressing the gene. G) Representation of CXCL signaling networks in APM‐treated and untreated samples, with circle size indicating cell group size and edge width representing communication probability. H) The significant ligand‒receptor pairs that contribute to the signals sent from tumor cells, fibroblasts, chondrocytes, pericytes and osteoclasts to neutrophils. The dot color and size indicate the calculated communication probability and *p* value, respectively. (H) Immunohistochemical staining of CXCR2 in human samples of adjacent kidney tissue, primary kidney tumor tissue and paired metastatic lesion tissue of bone metastatic RCC. N: adjacent; T: tumor; M: metastasis. I) Representative BL images showing the combination therapy of APM and SB225002 further reduces the tumor burden of bone metastasis in mice compared to APM treatment alone at multiple time points. (control group, *n* = 14; APM‐treated group, *n* = 14; APM+SB225002 group, *n*=12;). The error bars indicate the means ± SDs. The *p* values were determined by Student's *t* test; *p < 0.05, **p < 0.01, and ***p < 0.001.

Given that accumulating evidence from preclinical and clinical studies demonstrating the dual roles of neutrophils in tumor growth and metastasis,^[^ ^25^ ^]^ and that observed activation of pathway regulating neutrophil‐mediated cytotoxicity in APM‐treated cells (Figure 4C), we investigated the mechanism by which APM affects neutrophils. We subclustered extracted neutrophils into four subclusters based on marker expression: Neu1 (*Csf3r*+*Retnlg*+), Neu2 (*Lgals3*+*Ifitm6*+), Neu3 (*Icam1*+*Cxcl2*+), and Neu4 (*Top2a*+*Mki67*+) (**Figure** **5**A–C; Figure S7A, Supporting Information). Direct comparison of APM‐treated and APM‐naïve neutrophils revealed that neutrophil‐mediated cytotoxicity was upregulated in APM‐treated neutrophils (Figure 5D; Figure S7B, Supporting Information), while the regulation of neutrophil degranulation, regulation of neutrophil chemotaxis, TGF‐β1 production, NIF NK kappa signaling, and IL‐6 production were upregulated in APM‐naïve neutrophils (Figure 5D; Figure S7C, Supporting Information). Tumor‐associated neutrophils (TANs) can be phenotypically classified into proinflammatory N1 and anti‐inflammatory N2 subpopulations, which play distinct roles in tumor progression.^[^ ^26^ ^]^ Cell scoring further showed that the N1‐like neutrophil phenotype was highly activated in APM‐treated cells with downregulation TGF‐β signaling, while APM‐naïve cells exhibited the N2‐like neutrophil phenotype (Figure 5E). APM‐treated cells highly expressed N1 neutrophil‐associated genes, while low expression of N2 neutrophil‐associated genes compared to APM‐naïve cells (Figure 5F). N1 neutrophils are known for their anti‐tumor characteristics,^[^ ^27^ ^]^ whereas N2 neutrophils exhibit characteristics that enhance tumor growth and support tumor metastasis.^[^ ^28^ ^]^ Additionally, neutrophils among APM‐treated cells exhibited significant downregulation of genes associated with tumor metastasis, such as *S100a8*, *S100a9*, *Mmp8*, *Mmp9*, *Ifitm1*, *Ifitm2*, and *Ifitm3*, and neutrophil migration factors, such as *Cxcr2* (Figure S7D, Supporting Information).

Further investigating revealed that *Cxcr2* was exclusively expressed in neutrophils among APM‐naïve cells (Figure S7E, Supporting Information). High expression of the ligands of *Cxcr2*, such as *Cxcl1*, *Cxcl2*, *Cxcl3*, and *Cxcl5*, was observed mainly in tumor cells, fibroblasts, chondrocytes, pericytes and osteoclasts among APM‐naïve cells (Figure S7E, Supporting Information). Consistent with these findings, studies have demonstrated that the CXCL‐CXCR2 signaling axis promotes tumor growth, the homing of mature neutrophils into tissues, and invasiveness.^[^ ^29^ ^]^ These results suggest that APM‐naïve tumor cells enhance neutrophil infiltration to promote bone metastasis by upregulating the expression of *Cxcr2* and activating the Cxcl‐Cxcr2 signaling axis. To confirm the increased neutrophil recruitment in the APM‐naïve cell population, we used CellChat to identify putative signaling between neutrophils and other cell populations via known receptor‒ligand pairs.^[^ ^30^ ^]^ The results were consistent with the genes expression analysis, showing strong interaction between neutrophils with other cell types, including tumor cells, fibroblasts, chondrocytes, pericytes, and osteoclasts, under CXCL signaling pathway in APM‐naïve cells (Figure 5G; Figure S7E, Supporting Information). in contrast, these interactions were significantly weakened in APM‐treated cells (Figure 5G). Immunohistochemical analysis showed that neutrophil infiltration was lower in APM‐treated mice than in APM‐naïve mice (Figure S7F, Supporting Information). Importantly, neutrophil abundance was confirmed by immunohistochemistry in sections from adjacent kidney tissue, primary kidney tumors and paired metastatic lesions in human samples (Figure 5H). Furthermore, neutrophil abundance was significantly associated with the expression of *CXCL1*, *CXCL2*, *CXCL3*, and *CXCL5*, as well as CXCL‐CXCR2 signaling in ccRCC metastasis samples from the TCGA‐KIRC cohort (Figure S7G, Supporting Information), consistent with the findings in our current study. Moreover, CXCL‐CXCR2 signaling and neutrophil abundance were increased in patients with metastasis in the TCGA‐KIRC database (Figure S7H, Supporting Information). Thus, these results suggest that blockade of the CXCL‐CXCR2 axis by APM may reduce the risk of renal cancer metastasis. Next, we expect to further observe the effects of combination treatment with a *CXCR2* antagonist and APM. In this study, a selective and non‐peptide *CXCR2* antagonist, SB225002, was chosen to block CXCL/CXCR2 interaction. The in vivo results highlighted that the combination of SB225002 and APM could further inhibit the progression of bone metastasis (Figure 5I), which may be a promising treatment strategy for bone metastatic RCC.

## 3 Discussion

The growth of metastases in distant organs remains the major cause of cancer mortality. The development of bone metastasis is a multistep process, and it is now widely appreciated that the interactions between cancer stem cells and the metastatic niche play an integral role in the successful initiation of metastatic growth.^[^ ^31^ ^]^ Numerous reports have shown that fibrin is present in the connective tissue stroma in human malignant tumors,^[^ ^32^ ^]^ that fibrin and fibrinogen promote the survival and metastatic potential of circulating tumor cells,^[^ ^33^ ^]^ that fibrin–fibronectin complexes promote lung metastasis of melanoma,^[^ ^34^ ^]^ and that fibrinogen depletion inhibits pulmonary tumor formation in wild‐type mice.^[^ ^35^ ^]^ In this study, using 3D soft fibrin gels (purified fibrinogen activated by thrombin),^[^ ^14^ ^]^ we generated cancer stem cell‐like cells from human kidney cancer cells A498, Caki‐1 and mouse kidney cancer Renca cells, after 3D‐cultured for 5 days, these cells grew into a spheroid‐like morphology and upregulation of a series of stem cell markers, exhibited cancer stem‐like cell features. It is important to note that not all stem cell markers show elevated expression in tumor cells following 3D culture, which may be associated with variations in genetic backgrounds, cellular heterogeneity, and environment‐dependent expression differences of stem cell markers. Meanwhile, RNA‐seq also verified that stem cell related pathways were upregulated such as HIF, Notch signaling pathway components. These results suggested that in a 3D‐culture system, cancer cells showed more stem cell‐like characteristics and behaved more like cancer stem cells. Cancer stem cells are broadly accepted to be a potential strong driving force for metastasis, a property associated with their capacity to undergo EMT, their cancer stem cell‐like properties, and their ability to escape immunosurveillance.^[^ ^15^ ^]^ Consistent with these findings, 3D‐cultured Renca cells efficiently initiated bone metastatic outgrowth when injected through the CA in the mouse model which was more efficient than 2D‐cultured cells. The use of 3D fibrin gels in this study provides a more physiologically relevant model for studying renal cancer metastasis. Unlike traditional 2D cultures, 3D cultures better mimic the in vivo tumor microenvironment, allowing for the study of cancer stem cell properties and their role in metastasis. The upregulation of stem cell markers and signaling pathways in 3D‐cultured cells suggests that these cells are more aggressive and have a higher metastatic potential. The present approach not only provides a useful platform for understanding the mechanisms underlying the tumorigenicity of cancer stem cells but also opens a new avenue in research on metastatic outgrowth that considers interactions between cancer stem cells and their niche. This finding is crucial for understanding the early stages of metastasis and identifying potential therapeutic targets.

Interestingly, we observed that the antioxidant action of the vitamin C pathway was significantly suppressed in 3D‐cultured renal cancer cells, as assigned by IPA (Figure 2A), suggesting that vitamin C supplementation may specifically target cancer stem cells and inhibit metastasis. Indeed, we found that vitamin C treatment inhibited the proliferation of cancer stem cell‐like cells in vitro and blocked bone metastatic outgrowth in vivo. Consistent with these findings, previous studies, as well as our current study, showed that vitamin C exhibits a killing effect on certain types of cancer stem cells.^[^ ^11^, ^36^ ^]^ Lv et al. found that pharmacological vitamin C could preferentially kill cancer stem cells in hepatocellular carcinoma via intravenous injection, resulting in ROS generation, DNA damage and ATP depletion.^[^ ^37^ ^]^ It has been found that vitamin C as a prooxidant at the pharmacological dose (mM), increasing ROS in many types of cancers such as breast cancer and colorectal cancer.^[^ ^38^ ^]^ However, at physiological doses (µM), it may act as an antioxidant by increasing 5hmC levels through DNA methylation.^[^ ^10^, ^39^ ^]^ Our recent study also revealed that vitamin C at a low concentration can kill prostate cancer stem‐like cells and induce apoptosis by specifically increasing 5hmC modification.^[^ ^11^ ^]^ Additionally, in cancer cell metabolism, Vitamin C has the ability to inhibit hypoxic glycolysis through *GAPDH* inactivation or promoting the degradation of *HIF‐1*.^[^ ^40^ ^]^ While most previous studies have focused on the role of vitamin C in targeting tumor cells. recent research has shown vitamin C also targets the tumor microenvironment to exert anticancer activity. It may reverse the immunosuppressive tumor microenvironment to the immunoreactive one by increasing the infiltration of T cells, inducing apoptosis of infiltrating M2‐type TAM, or attenuating neutrophil extracellular trap formation.^[^ ^41^ ^]^ Vitamin C enhances anti‐tumor immune responses by inhibiting *STAT1* dephosphorylation in tumor cells through a post‐translational modification mechanism.^[^ ^42^ ^]^ Furthermore, vitamin C may inhibit tumor angiogenesis and promote vessel normalization by downregulating *VEGF* mRNA expression.^[^ ^43^ ^]^ It also plays a crucial role in regulating the stiffness of ECM by altering the gene expression profile of stromal cells.^[^ ^44^ ^]^ Unlike previous researches, in our study, we found that vitamin C could directly kill tumor cells by inducing cell cycle arrest and remodeling the tumor microenvironment to inhibit the growth of bone metastases, effects that have not been identified.

We next examined the mechanism of vitamin C inhibits metastatic outgrowth and their niche. We profiled and compared the single‐cell transcriptome profiles between vitamin C‐naïve and vitamin C‐treated bone metastatic carcinoma cells and found that vitamin C affected not only malignant cells but also stromal cells and immune cells by suppressing osteoclast differentiation and enhancing neutrophil‐mediated cytotoxicity. Inflammatory cytokines, such as IL‐6 and matrix metalloproteinases in both tumor and stromal cells play important roles in modulating the bone metastatic process of tumor cells.^[^ ^16^, ^45^ ^]^ In this study we found that IL‐6 and MMP‐related pathways were inhibited in vitamin C treated group mice. Moreover, accumulating evidence has shown the positive effects of vitamin C on bone health, such as activating osteoblastogenesis and inhibiting osteoclastogenesis,^[^ ^12^, ^46^ ^]^ potentially mitigating tumor‐induced aggressive osteolysis, which was also verified by the results of analysis and the experiment of osteoclast differentiation in this study. Since bone metastases in RCC are mainly osteolytic, leading to bone destruction, vitamin C may function as a protective factor against bone metastasis. As for whether vitamin C has similar effects on all osteolytic bone metastases, including breast cancer, and its therapeutic effect on prostate cancer characterized by osteoblastic bone metastases, further in‐depth research is still needed. In addition, neutrophils may act as inadvertent accomplices of tumor cells in cancer progression and metastasis.^[^ ^47^ ^]^ In animal models of alcoholic liver disease, vitamin C dramatically attenuated ethanol‐mediated liver disease by decreasing the infiltration of neutrophils.^[^ ^48^ ^]^ Importantly, we also found a higher neutrophil abundance in patient samples of bone metastases than in the paired samples of primary kidney tumors and adjacent tissues. In the mouse model of bone metastasis, treatment with vitamin C decreased neutrophil infiltration. Overall, to our knowledge, we are the first to generate single‐cell transcriptomes showing vitamin C‐related differences in bone metastases and revealed the diverse functions of vitamin C as an antitumor agent in reprogramming malignant cells, stromal cells and monocytic cells in the bone metastatic microenvironment. The tumor microenvironment plays a critical role in the progression of bone metastasis. Our findings show that vitamin C not only affects malignant cells but also reprograms the stromal and immune components of the microenvironment. The suppression of osteoclast differentiation and enhancement of neutrophil‐mediated cytotoxicity highlight the importance of targeting the microenvironment in cancer therapy. By modulating the behavior of stromal cells and immune cells, vitamin C can create a less favorable environment for cancer growth and metastasis. Neutrophils are a double‐edged sword in cancer progression, with both pro‐tumor and anti‐tumor roles. Our study shows that vitamin C can shift the balance toward a more anti‐tumor phenotype by reducing the infiltration of neutrophils and downregulating pro‐metastatic signals. This finding is particularly significant given the high abundance of neutrophils in bone metastases and their potential to promote cancer progression. By targeting neutrophils, vitamin C may help to prevent the establishment of a metastatic niche and reduce the risk of further metastatic spread.

Additionally, vitamin C deficiency is associated with immune system impairment,^[^ ^49^ ^]^ and immune cells are important for antitumor responses. Thus, recent in vivo murine studies demonstrated that vitamin C contributes to the antitumor treatment response by reprogramming the immune system and enhancing the effect of immune checkpoint inhibitor therapy.^[^ ^41^, ^50^ ^]^ Moreover, studies have shown that vitamin C can also be used as an adjuvant to enhance the effect of chemotherapy.^[^ ^51^ ^]^ Considering the anticancer function of vitamin C, establishing new strategies for its use, especially in combination therapies, to inhibit cancer progression in various types of cancer is very important. Therefore, according to the analysis results, APM could significantly decrease the expression of *Cxcr2* in neutrophils, we used the *CXCR2* antagonist SB225002 in combination with APM and found it could further inhibit the growth of bone metastasis, which may be an ideal treatment strategy enhancing the inhibitory effects of vitamin C for bone metastatic RCC. The combination of vitamin C with the *CXCR2* antagonist SB225002 represents a promising therapeutic strategy. This combination not only enhances the inhibitory effects of vitamin C on bone metastasis but also provides a synergistic approach to targeting multiple aspects of cancer progression. The use of combination therapies is increasingly recognized as a more effective approach to cancer treatment, as it can overcome the limitations of single‐agent therapies and address the heterogeneity of cancer cells and their microenvironment. In summary, based on the findings in this study, we provide proof of concept for the use of vitamin C to treat bone metastatic RCC. Our observations provide important hypothesis‐generating insights that may spur new clinical trials for renal cancer. The findings highlight the importance of targeting cancer stem cells and their niche in cancer therapy. The comprehensive and high‐resolution profile of the antitumor effects of vitamin C in the bone metastatic microenvironment elucidated in this work not only highlights the potential of vitamin C as a therapeutic agent but also suggest that vitamin C, alone or in combination with other agents, may be a valuable addition to the current treatment options for renal cancer patients. Future studies should focus on further elucidating the mechanisms of action of vitamin C and validating its therapeutic potential in clinical settings.

## 4 Experimental Section

### Animals and Cell Lines

Four‐week‐old male BALB/c mice were purchased from Charles River. Animal experiments were performed in accordance with the NIH Guidelines for the Care and Use of Laboratory Animals with the approval of the Animal Experiment Committees of Peking University First Hospital (No. J202006). The RCC cell lines used in this study were cultured as follows: Renca cells were cultured in RPMI‐1640 medium (Gibco), and A498, Caki‐1, RAW264.7 cells were cultured in DMEM (HyClone); all media contained 10% fetal bovine serum (FBS; BI), 100 U ml^−1^ penicillin, and 100 µg ml^−1^ streptomycin, and all cell lines were cultured in a humidified atmosphere with 5% CO_2_ at 37 °C.

### 3D Fibrin Gel Culture of Renal Cancer Cells

The 3D fibrin gel culture system was used according to a method described in a previous study.^[^ ^14^ ^]^ Briefly, fibrinogen (Sea Run Holdings) was dissolved in T7 buffer (pH 7.4; 50 mm Tris, 150 mm NaCl) to a concentration of 20 mg ml^−1^. The proper volumes of the fibrinogen solution and the cell solution were mixed, resulting in a final fibrinogen concentration of 1 mg ml^−1^ (90 Pa). A total of 250 µl of the cell/fibrinogen mixture was seeded into each well of a 24‐well plate with 5 µl of thrombin (0.1 U µl^−1^) and was then incubated at 37 °C for 1 h. Finally, 1 ml of DMEM containing 10% FBS and antibiotics was added to the wells. After culture for 1, 3, 5, and 7 days, the diameter of the cell spheroids was measured.

### RNA Extraction and qPCR Analysis

Total RNA was extracted following the manufacturer's protocol by TRIzol reagent (Invitrogen). The RevertAid First Strand cDNA Synthesis Kit (Invitrogen) was used to reverse transcribe 1 µg of total RNA to cDNA. The KAPA SYBR FAST Universal qPCR Kit (Applied Biosystems) was used to perform RT–qPCR on a 7500 Fast Real‐Time PCR system (Applied Biosystems). The primer sequences used for qPCR are listed in Table S4 (Supporting Information).

### Bone Metastasis Models

For CA injection, spheroids were harvested and digested into single cells before injection. Renca/luc cells (1×10^5^ cells per male BALB/c mouse) cultured under conventional 2D rigid dish or 3D fibrin gel conditions and suspended in 100 µl of PBS were injected into mice via the CA using a 29G syringe needle within 3s.

### Treatment Experiments

APM (Sigma‒Aldrich; cat# A8960) formulated in sterile water was administered as a single agent to mice in the treatment group by intraperitoneal injection once daily at a dose of 0.5 g kg^−1^ body weight. APM treatment was begun 7 days before injection of cancer cells and continued until the observation endpoint. Mice in the control group were treated with normal saline under the same conditions. For combination treatment group received 5 mg kg^−1^ of SB225002 intraperitoneally each day with 0.5 g kg^−1^ APM. SB225002 (MedChemExpress; cat# HY‐16711) was dissolved in a 10% dimethyl sulfoxide (DMSO), 40% polyethylene glycol 400 (PEG300), 5% polysorbate 80 (Tween 80), and 45% Saline.

### In Vivo BL Imaging

BL images of tumor‐bearing mice were acquired with an IVIS Spectrum imaging system 15 min after intraperitoneal injection of D‐luciferin (50 mg kg^−1^). The following conditions were used for image acquisition: open emission filter, exposure time = 60s. The BL images were analyzed by Living Image 4.3 software (PerkinElmer) specialized for the IVIS system.

### Ex Vivo BL Imaging

A mouse was sacrificed immediately after in vivo BL imaging, and the major organs were removed. BL images of the organs were acquired with the following imaging conditions: open emission filter, exposure time = 30s. The BL images were analyzed by Living Image 4.3 software (PerkinElmer) specialized for the IVIS system.

### Histological Analysis

An isolated hind limb bone was fixed in 70% ethanol for 48 h, decalcified in 10% EDTA for 2 weeks, processed, and embedded in paraffin. Bone sections (10 µm thickness) were then stained with HE.

### Immunohistochemistry

A primary antibody specific for CXCR2 (YT5397, ImmunoWay, 1:800) was used. Formalin‐fixed, paraffin‐embedded tissue sections (5 µm) were deparaffinized and rehydrated. Antigen retrieval was carried out using 10 mm sodium citrate (pH 6.0). Endogenous peroxidase activity was blocked by incubation with 0.3% hydrogen peroxide for 10 min and 5% BSA in PBS for 1 h. Slides were incubated first with a primary antibody overnight at 4 °C and then with an HRP‐conjugated secondary antibody (PV‐9001, ORIGENE) at room temperature (30 min). Diaminobenzidine (DAB) was used as a chromogen, and sections were counterstained with hematoxylin. Representative fields were selected from these slides.

### Osteoclast Differentiation Validation

The RAW264.7 cells were seeded into the 6‐well plates overnight with two groups. They were then induced with the addition of a medium including 50 ng ml^−1^ M‐CSF and 100 ng ml^−1^ RANKL and with/without 0.5 mm APM. The cell growth morphology was observed under microscopy. After 7 days of incubation, the osteoclasts were verified by TRAP stain using the TRAP staining kit (Solarbio, China) according to the instructions.

### Dissociation of Tissues into Single Cells

The tumor and surrounding bone tissue regions of all samples were processed immediately after resection. Fresh samples were placed in an ice solution containing Hank's balanced salt solution and 1% antifungal antibody and transferred the tissue into 4 °C refrigerator for storage within 20 min. Then the sample was token and mince into 1 mm^3^ pieces. Resolvase mix (Collagenase II C8150 Solarbio, DNase I SIGMADN25, FBS2%Gibco 10100139) was used to digested sample pieces into single cells. In short, Resolvase mix was added into samples pieces. Using gentleMACS Dissociator to dissociated tissues. Then filter the suspension with filter membrane (Miltenyi Biotec 130‐110‐916). Next, the suspended cells were centrifuged at 300 g for 10 min. After removing the supernatant, red blood cell lysis buffer (Solarbio R1010) was used to resuspend the cell pellets for 3 min. The cell pellets were washed twice with 1 × PBS. To facilitate comparative analysis, single cells derived from tumor and surrounding bone tissue of the same sample were pooled together. Meanwhile, cell viability would be assessed using Hochest staining, then using BD FACSAria Fusion cell sorter to isolate live cells. By following the manufacturer's protocol, the Single Cell 3′ Library and Gel Bead Kit V3.1 (10x Genomics, 1 000 075) and Chromium Single Cell B Chip Kit (10x Genomics, 1 000 074) were used to prepare barcoded scRNA‐seq. Paired‐end 150 bp reads were then generated by sequencing the libraries on the Illumina NovaSeq6000 platform (performed by CapitalBio Technology, Beijing).

### Quality Control and Correction of scRNA‐seq Data

CellRanger v6.1.2 (10X Genomics) and the mouse mm10 reference transcriptome was used to demultiplex and process the single‐cell RNA‐seq raw FASTQ files for all samples, and the DoubletFinder R package (version 2.0.3)^[^ ^52^ ^]^ was then used with the default parameters to computationally predict and filter doublets in each sample. After removal of doublets, the Seurat R package (version 4.0.5)^[^ ^53^ ^]^ was used to analyze the output‐filtered gene expression matrices. In brief, for each cell, two quality parameters: the percentage of unique molecular identifiers (UMIs) derived from the mitochondrial genome and the number of total genes expressed were calculated. Low‐quality cells with a mitochondrial DNA percentage of more than 25% and fewer than 200 or more than 3000 total expressed genes were removed.

### Batch Effect Correction, Normalization, Sample Integration, and Cell Clustering

First, each sample was normalized using the SCTransform approach.^[^ ^54^ ^]^ Then, to determine the intersample anchors for sample integration, the FindIntegrationAnchors function was used with the top 3000 highly variable genes identified with the SelectIntegrationFeatures function across the samples. Then the IntegrateData function was used to obtain a combined and a batch‐corrected expression matrix. Principal component analysis (PCA) was carried out on this batch‐corrected expression matrix. The top 50 principal components were used to generate a shared nearest neighbor (SNN) cell plot, which was then used for clustering with the Louvain algorithm (resolution = 0.6) to obtain an initial clustering result with the FindClusters function. The top 50 principal components were mapped into two dimensions using the Uniform Manifold Approximation and Projection (UMAP) algorithm to visualize the cells.

### Differential Gene Expression Analysis and Major Cell Type Annotation

The *FindMarkers* or *FindAllMarkers* function with the parameter MAST was used to find markers for each of the identified clusters or subtypes. Genes with adjusted p < 0.05 were considered significantly differentially expressed. Then, cell types were annotated on the basis of the expression of classical markers for particular cell types.

### Identification of Malignant Cells

Normal cells from samples obtained from healthy mice, as published by Tsukasaki M. et al.,^[^ ^55^ ^]^ were used as control cells. The count matrices of the control cell data were downloaded from GEO under accession number GSE147174. Epithelial cells with high expression levels of Krt8 and Krt18 were considered as malignant cells for further analysis.

### Cell Cycle Analysis

Cell cycle phase assignment was performed based on the normalized expression matrix. It was found that cells were noncycling or were in the G1, S or G2/M phase by using the *cyclone* function in the R package scran and the expression level of MKI67.

### GSEA

GSEA was performed using the fgsea v1.16.0 R package to analyze differentially enriched pathways in different subclusters. For a given pairwise GSEA comparison, the log2 (fold change) in the average expression level between the groups was used as a ranking metric. GSEA was performed using Hallmark, KEGG, and GO Biological Process gene sets from the msigdbr v7.2.1 R package or selected gene sets curated from the published literature.^[^ ^24^, ^26^, ^56^ ^]^ Gene sets with p < 0.05 and FDR < 0.25 were considered significantly enriched.

### Cell Scoring

To quantitatively evaluate the degree to which individual cells in the cell populations expressed a certain predefined gene set (for example, the TIM or TAM signature), the *AddModuleScore* function was utilized to calculate the cell scores for each population. The gene signatures utilized for scoring TIM, TAM, N1, and N2 were derived from previous studies.^[^ ^24^, ^26^, ^56^, ^57^ ^]^

### Pseudotime Trajectory Analysis of Differential Gene Expression

To determine and establish the potential cell trajectories during osteoclast differentiation, the Monocle2 (version 2.22.0) R package was applied, which includes monocytes, preosteoclasts, macrophages, and osteoclasts. The gene‐cell matrix of UMI counts was provided as the input to Monocle, and then, the *newCellDataSet* function was employed to create a CellDataSet with the parameter expressionFamily = negbinomial.size. Then the *differentialGeneTest* function was used to identify genes significantly differentially expressed over time.

### Cell‒Cell Interaction Inference

To analyze cell‒cell interactions between different cell types, CellChat (http://www.cellchat.org/) was applied to identify significant ligand‒receptor pairs within APM‐treated and APM‐naïve samples from the scRNA‐seq data.

### Quantification and Statistical Analysis

All statistical analyses were performed using R software (version 4.1.1). For comparison of the signature scores between different cell groups and bulk RNA‐seq sample groups, a two‐sided Wilcoxon rank‐sum test was used. Detailed descriptions of the statistical tests performed for the individual analyses are provided in the figure legends and the Experimental Section.

## Conflict of Interest

The authors declare no conflict of interest.

## Author Contributions

J.Z., Q.Z., and G.L. contributed equally to this work. L.Z, W.C., X.L., Y.S. conceived the study. J.Z., Q.Z., W.C., and L.Z. designed the experiments. J.Z., Q.Z., G.L., Y.W., J.L., P.W., J.Q., Y.L., S.H., Y.G., N.F., Y.W., Y.M., M.Z., and Y.S. performed the experiments and analyzed the data. W.C., J.Z., Q.Z. and Y.S. wrote the paper.

## Supporting information



Supporting Information

Supplemental Table 1

Supplemental Table 2

Supplemental Table 3

Supplemental Table 4

## Data Availability

Data have been deposited in the Genome Sequence Archive for Human (GSA‐Human) under accession HRA003215^[^ ^58^ ^]^ (https://ngdc.cncb.ac.cn/gsa‐human). The data that support the findings of this study are available from the corresponding author upon reasonable request.
