# Supplementary material for: Single‐Cell Analysis Reveals that Vitamin C Inhibits Bone Metastasis of Renal Cancer via Cell Cycle Arrest and Microenvironment Remodeling
Source: Adv Sci (Weinh). 2025 May 28;12(30):e01011. doi: 10.1002/advs.202501011 (PMC12376498; doi:10.1002/advs.202501011)
Supplement: Supplementary file 1 — Supporting Information [file ADVS-12-e01011-s004.docx]

**
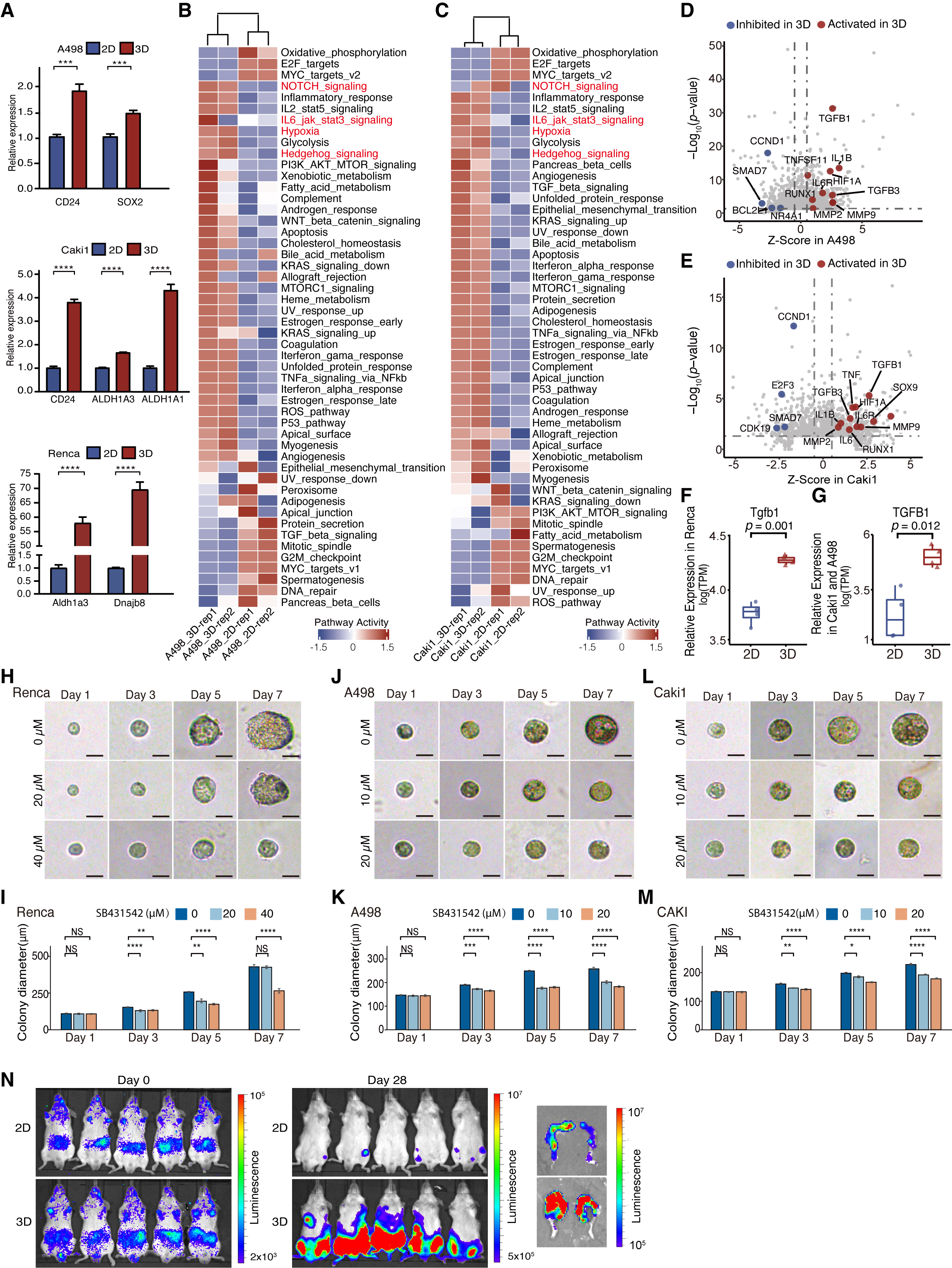
**

**Figure S1. TGF-β1 was upregulated in 3D-cultured renal cancer cells, and the inhibitory effect of the TGF-βR inhibitor on spheroid growth**

(A) Bar graphs depicting the mRNA levels of stem cell markers (*CD24*, *SOX2*, *ALDH1A3*, *ALDH3A1* and *DNAJB8*) in 2D and 3D cultures of Renca, A498, and Caki-1 cells.

(B, C) Heatmap of transcriptome analysis results indicating stem cell-associated and malignancy-associated signaling pathways upregulated in 3D-cultured A498 (B) and Caki-1 (C) cells.

(D, E) Volcano plot showing the results of IPA to predict differences in upstream regulators between the 3D-cultured and the 2D-cultured group of A498 (D) and Caki-1 cells (E).

(F) Expression of *Tgf-β1* in a mixed group with 3D cultured Renca cells compared with the 2D group.

(G) Expression of *TGF-β1* in a mixed group with 3D cultured A498 and Caki-1 cells compared with the 2D group.

(H, I) The colony size of 3D cultured Renca cells treated with different concentrations of the SB431542. The error bars indicate the means ± SDs. The *p* values were determined by Student’s *t* test, **p*< 0.05, ***p*<0.01, ****p*<0.001, *****p*<0.0001. Bar, 50μm.

(J, K) The colony size of 3D cultured A498 cells treated with different concentrations of SB431542. The error bars indicate the means ± SDs. The *p* values were determined by Student’s *t* test, **p*< 0.05, ***p*<0.01, ****p*<0.001, *****p*<0.0001. Bar, 50μm.

(L, M) The colony size of 3D cultured Caki-1 cells treated with different concentrations of SB431542. The error bars indicate the means ± SDs. The *p* values were determined by Student’s *t* test, **p*< 0.05, ***p*<0.01, ****p*<0.001, *****p*<0.0001. Bar, 50μm.

(N) 2D and 3D cultured Renca/luc cells to establish a mouse model of bone metastasis (left) and *ex vivo* BL imaging intensity between the 3D group and the 2D group (right).


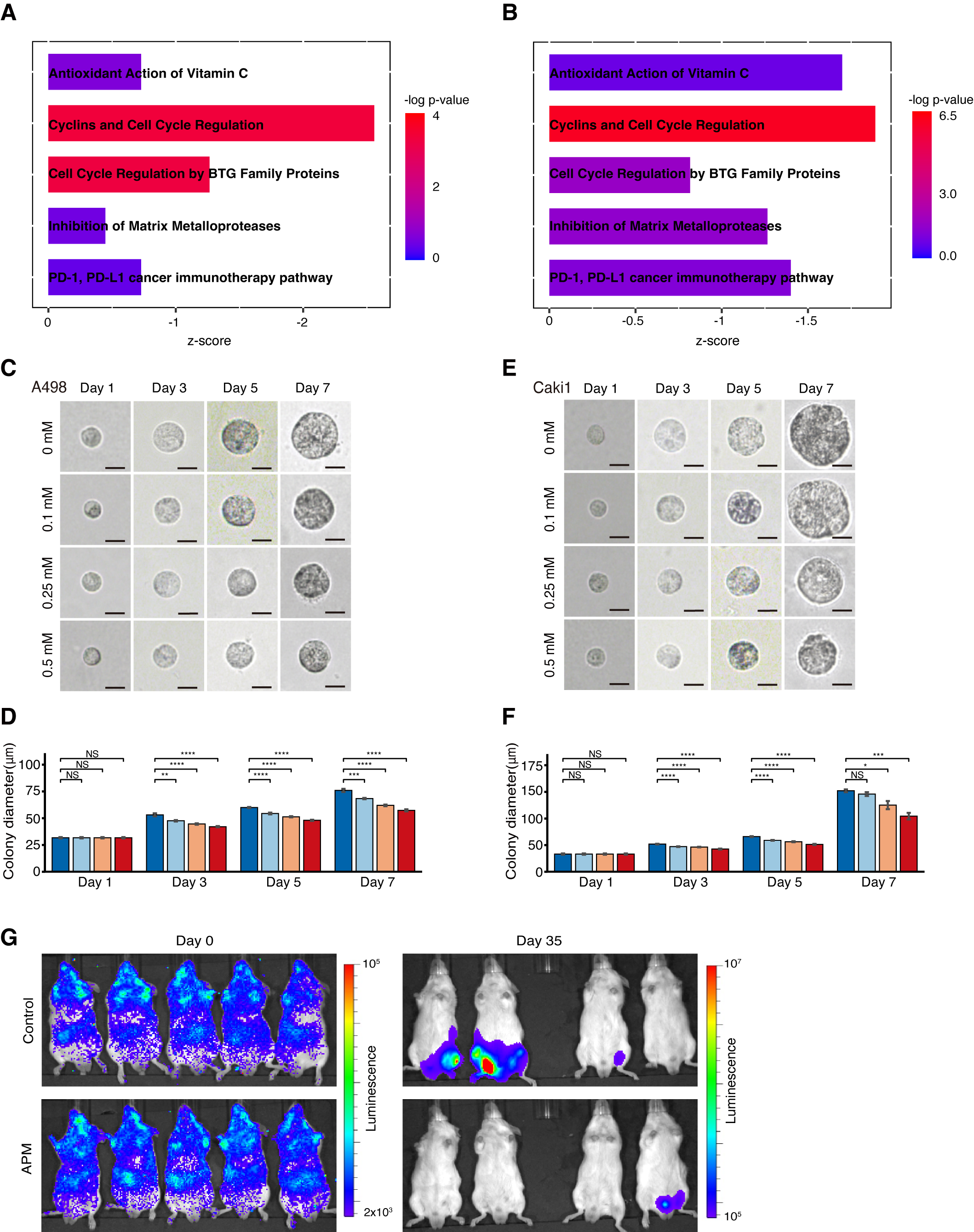


**Figure S2. APM inhibited the growth of 3D cultured A498 and Caki-1 cells *in vitro* and the progression of bone metastasis constructed by Renca cells.**

(A, B) IPA was performed to predict the negatively regulated pathways in the 3D-cultrued A498 (A) and Caki-1 (B) cells.

(C, D) APM inhibited the colony size of 3D-cultured A498 cells. Bar, 50μm. The error bars indicate the means ± SDs. The *p* values were determined by Student’s *t* test, **p*< 0.05, ***p*<0.01, ****p*<0.001, *****p*<0.0001. Bar, 50μm.

(E, F) APM inhibited the colony size of 3D-cultured Caki-1 cells. Bar, 50μm. The error bars indicate the means ± SDs. The *p* values were determined by Student’s *t* test, **p*<0.05, ***p*<0.01, ****p*<0.001, *****p*<0.0001. Bar, 50μm.

(G) APM inhibited the progression of the 3D cultured Renca bone metastatic model.

**
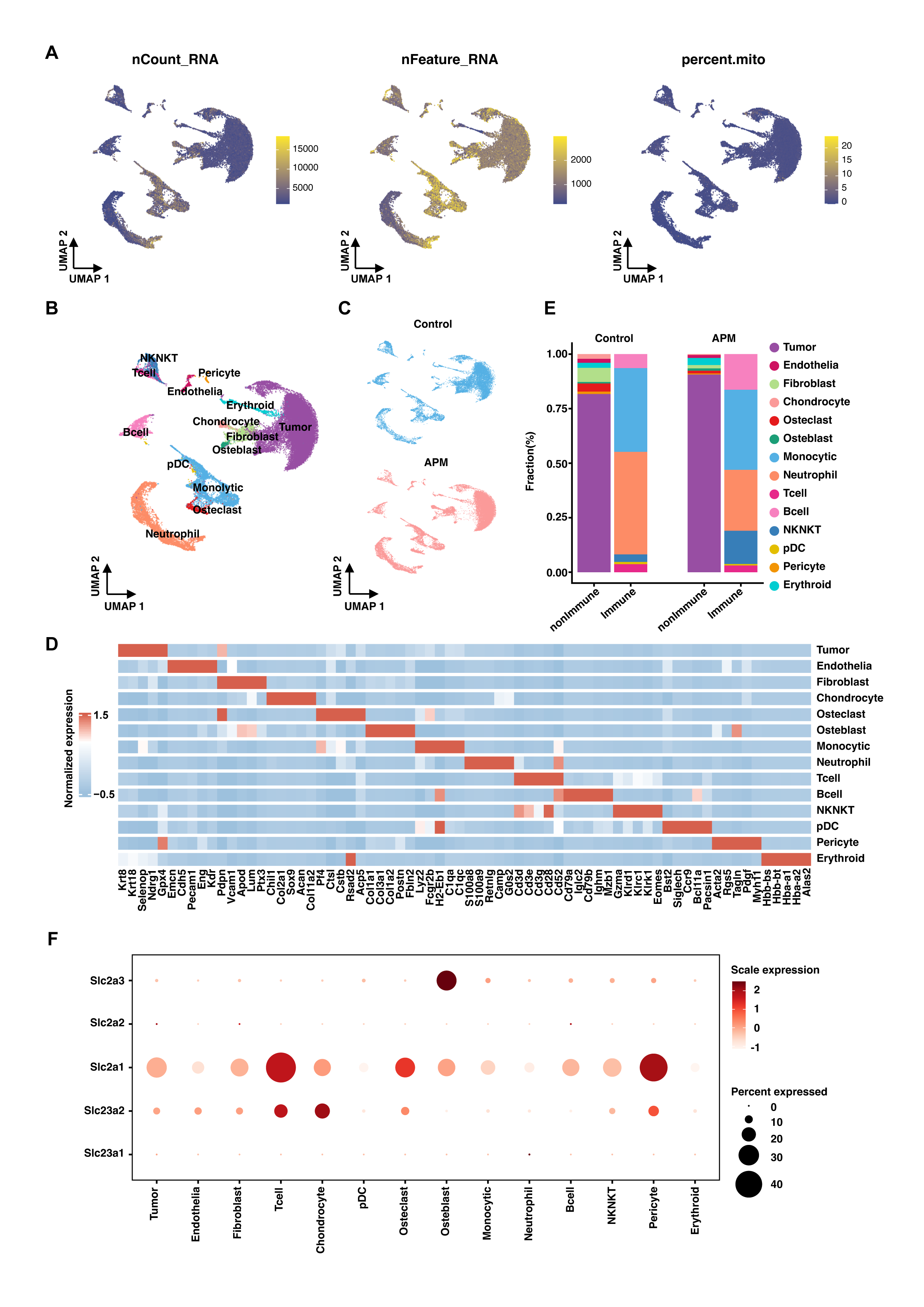
**

**Figure S3.** **scRNA-seq profiling of the tumor and tumor microenvironments treat with APM.**

(A) UMAP plots of cells colored by the number of genes detected, UMI RNA, and percentage of mitochondrial genes.

(B) UMAP plots showing cell populations identified from APM-treated and APM-naïve samples shows the formation of 14 main clusters with label names. Each dot corresponds to a single cell, colored according to cell type.

(C) UMAP plots showing the cell compositions of APM-treated and APM-naïve cells.

(D) Heatmap showing the expression of marker genes in the indicated cell types. The right bars label the clusters corresponding to specific cell types.

(E) Bar plot indicating the proportion of cells in tumor tissue of APM-treated and APM-naïve samples. Nonimmune cells and immune cells are shown in separate bars.

(F) Dotplot showing the expression of vitamin C receptor genes in specific cell types.

**
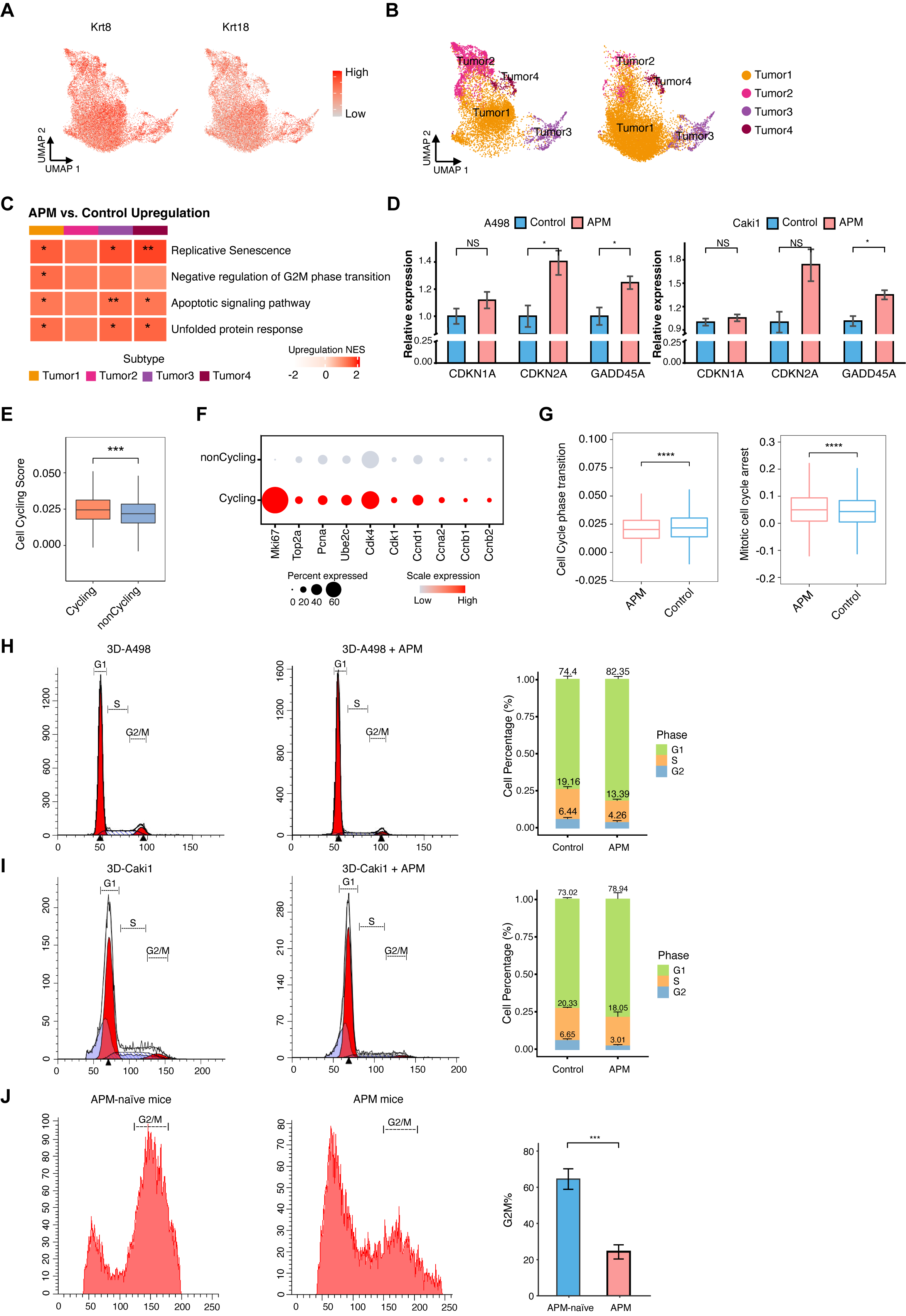
**

**Figure S4. APM-induced cell cycle arrest in malignant cells**

(A) UMAP plot showing the expression levels of Krt8 (left) and Krt18 (right), defined for all cell subtypes.

(B) UMAP plot showing malignant cell subtypes derived from APM-treated (left) and APM-naïve (right) cells, colored by cell subtype.

(C) Heatmap of GSEA normalized enrichment scores (NESs) indicating the pathways significantly enriched in tumor subtypes from APM-treated samples compared with APM-naïve samples.

(D) Cell cycle checkpoint markers in 3D cultured A498 (left) and Caki-1 (right) cells with and without APM were quantified by RT‒qPCR. Error bars represent the mean ± SD. The *p* values were determined by Student’s *t* test, **p*<0.05, ***p*<0.01, ****p*<0.001, *****p*<0.0001.

(E) Boxplot comparing cell cycling signature score distributions between the cycling and noncycling cells. The *p* value was determined by a two-sided Wilcoxon rank-sum test, **p*<0.05, ***p*<0.01, ****p*<0.001, *****p*<0.0001.

(F) Dotplot illustrating the expression levels of genes associated with cell cycle progression in cycling versus noncycling cells.

(G) Boxplots comparing cell cycle phase transition and mitotic cell cycle arrest signature score distributions in cycling cells partitioned by treatment status. The *p* value was determined by a two-sided Wilcoxon rank-sum test, **p*<0.05, ***p*<0.01, ****p*<0.001, *****p*<0.0001.

(H, I) Cell cycle distribution was assessed by flow cytometry in APM-treated and APM-naïve samples in A498 (H) and Caki-1(I) cells. Quantitation of the percentages of cells in each phase of the cell cycle. Experiments were repeated in triplicate, and the results are presented as the means.

(J) Cell cycle distribution was assessed by flow cytometry in APM-treated and APM-naïve mice tumor cells. Experiments were repeated in triplicate, and the results are presented as the means.


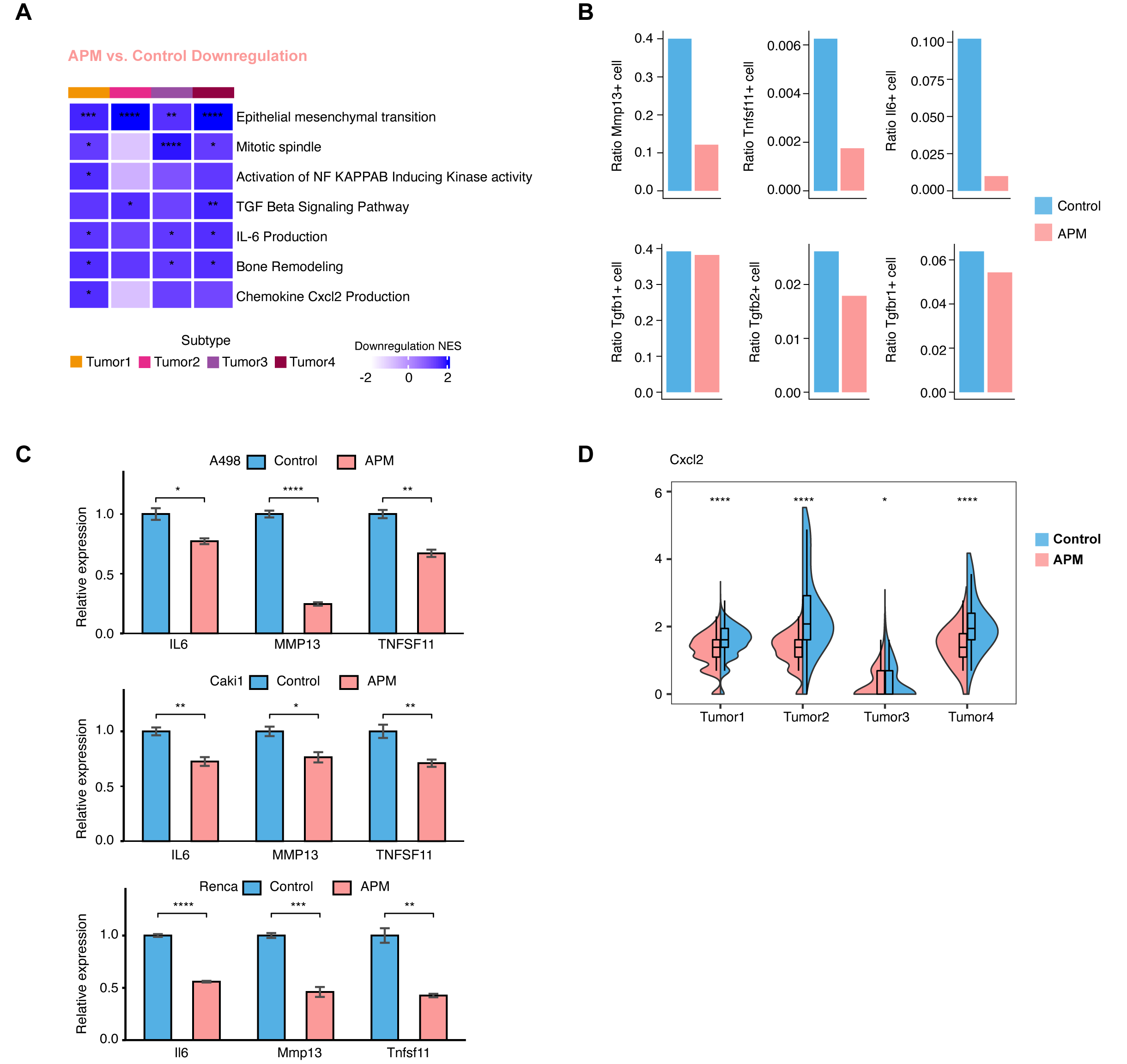


**Figure S5. APM inhibited the bone metastasis associated pathways of malignant cells.**

(A) Heatmap of GSEA NESs showing the pathways significantly enriched in tumor subtypes from APM-naïve samples compared with APM-treated samples.

(B) Bar plot illustrating the fraction of *Mmp13*^+^, *Tnfsf11*^+^, *Il-6*^+^, *Tgfb1*^+^, *Tgfb2*^+^ and *Tgfbr1*^+^ malignant cells in APM-treated and APM-naïve samples.

(C) The mRNA levels of *Il-6, Mmp13* and *Tnfsf11* in 3D cultured A498, Caki-1 and Renca cells with and without APM was quantified by RT‒qPCR. Error bars represent the mean ± SD. The *p* values were determined by Student’s *t* test, **p*< 0.05, ***p*<0.01, ****p*<0.001, *****p*<0.0001.

(D) Violin and box plots comparing the expression of Cxcl2 between APM-treated and APM-naïve cells. The *p* value was determined by a two-sided Wilcoxon rank-sum test. **p*< 0.05, ***p*<0.01, ****p*<0.001, *****p*<0.0001.


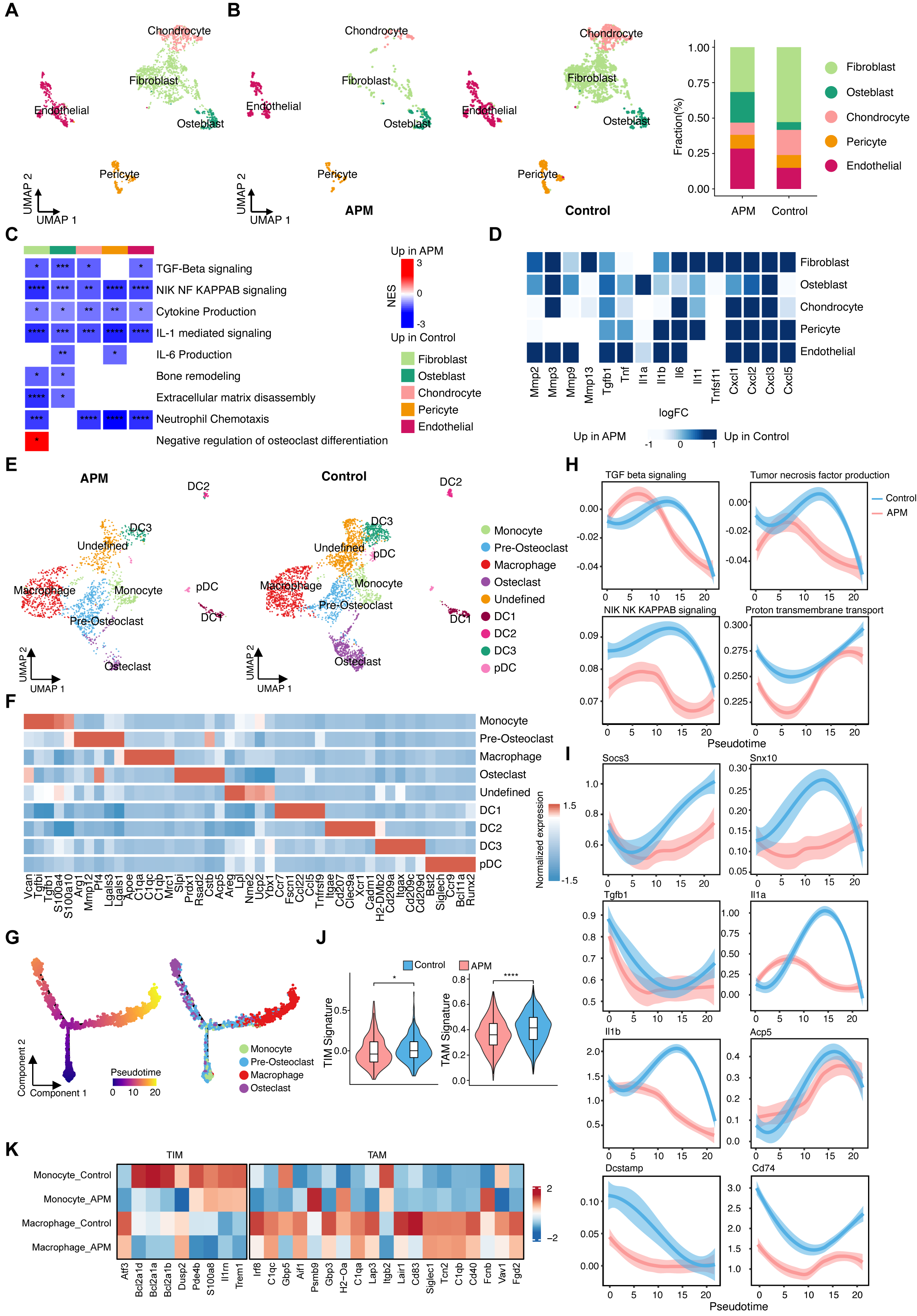


**Figure S6. Characteristics of osteoblasts and osteoclast in APM and APM-** **naïve groups.**

(A) UMAP of fibroblasts, osteoblasts, chondrocytes, pericytes and endothelial cells captured across all samples, colored and labeled by cell type.

(B) UMAP plot showing fibroblasts, osteoblasts, chondrocytes, pericytes and endothelial cells derived from APM-treated and APM-naïve cells, colored by cell subtype. And bar plots show cluster proportions grouped by APM treatment history.

(C) Heatmap of GSEA NESs indicating the pathways significantly enriched in fibroblasts, osteoblasts and chondrocytes from APM-treated samples or APM-naïve samples.

(D) Heatmap indicating the fold change in the expression of cytokine genes in comparisons of cells within each cluster from APM-naïve versus APM-treated samples.

(E) UMAP plot showing myeloid subtypes derived from APM-treated and APM-naïve cells, colored by cell subtype.

(F) Heatmap showing the expression of selected marker genes in myeloid subtypes.

(G) Pseudotime-ordered data of monocytes, preosteoclasts, macrophages and osteoclasts from APM-treated and APM-naïve samples. Cell subtypes are labeled by colors.

(H) Two-dimensional plots showing the expression scores for genes related to TGF-β signaling, tumor necrosis factor production, NIK NK kappa signaling and proton transmembrane transport in APM-treated (red) and APM-naïve (blue) samples, along with the pseudotime.

(I) Two-dimensional plots showing the dynamic expression of osteoclast differentiation-related genes along the pseudotime.

(J) Violin and boxplots comparing TIM and TAM signature score distributions between monocytes and macrophages, partitioned by treatment status. The *p* value was determined by a two-sided Wilcoxon rank-sum test. **p*< 0.05, ***p*<0.01, ****p*<0.001, *****p*<0.0001.

(K) Expression of TIM and TAM related marker genes in APM-treated and APM-naïve group.

**
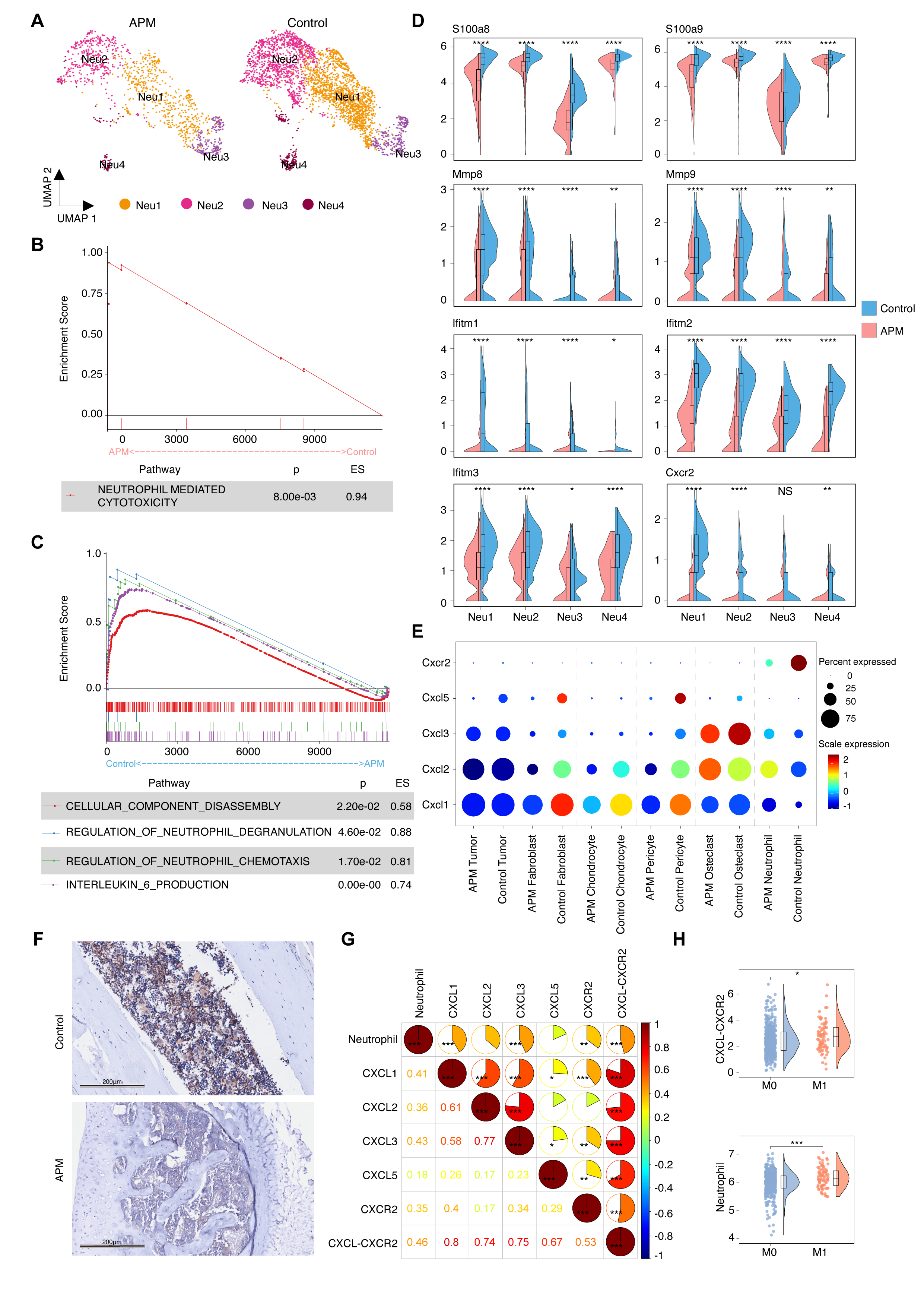
**

**Figure S7. APM actived neutrophil-mediated cytotoxicity and decreased neutrophil infiltration.**

(A) UMAP plot showing neutrophil subtypes derived from APM-treated and APM-naïve cells, colored by cell subtype.

(B) GSEA of neutrophil-mediated cytotoxicity signatures in neutrophils from APM-treated samples compared with APM-naïve samples.

(C) GSEA of cellular component disassembly, regulation of neutrophil degranulation, regulation of neutrophil chemotaxis and IL-6 production signatures in neutrophils from APM-naïve samples compared with APM-treated samples.

(D) Violin and boxplots comparing the expression of selected genes between each neutrophil subtype partitioned by treatment status. The *p* value was determined by a two-sided Wilcoxon rank-sum test, **p*<0.05, ***p*<0.01, ****p*<0.001, *****p*<0.0001.

(E) Dotplot showing the expression level and percentage of selected genes in different cell types between APM-treated and APM-naïve cells.

(F) Immunohistochemistry of APM-naïve tissue of bone metastasis (top) and mouse bone metastasis lesions treated with APM (bottom) stained for *Cxcr2*.

(G) Correlations between neutrophil abundance and *CXCL1, CXCL2, CXCL3, CXCL5, CXCR2* and CXCL-CXCR2 axis signaling in TCGA-KIRC metastasis samples.

(H) Violin plots depicting signature scores for CXCL-CXCR2 axis signaling and neutrophils, grouped by metastasis or non-metastasis status. The *p* values were determined by Student’s *t* test, **p*<0.05, ***p*<0.01, ****p*<0.001, *****p*<0.0001.
